# Post-translational regulation and proteolytic activity of the metalloproteinase ADAMTS8

**DOI:** 10.1016/j.jbc.2021.101323

**Published:** 2021-10-21

**Authors:** Salvatore Santamaria, Daniel R. Martin, Xiangyi Dong, Kazuhiro Yamamoto, Suneel S. Apte, Josefin Ahnström

**Affiliations:** 1 Department of Immunology and Inflammation, Imperial College London, London, UK; 2 Department of Biomedical Engineering, Cleveland Clinic Lerner Research Institute, Cleveland, Ohio, USA; 3 Institute of Life Course and Medical Sciences, University of Liverpool, Liverpool, UK

**Keywords:** ADAMTS, aggrecan, osteopontin, proteoglycan, TIMP, versican, ADAMTS, a disintegrin-like and metalloproteinase domain with thrombospondin motifs, α2M, alpha-2 macroglobulin, CR, cysteine-rich domain, Dis, disintegrin-like domain, DMEM, Dulbecco’s modified eagle’s medium, DTT, dithiothreitol, ECM, extracellular matrix, LC-MS/MS, liquid chromatography–tandem mass spectrometry, LRP1, low-density lipoprotein receptor-related protein 1, MEF, mouse embryonic fibroblast, MEM, minimum essential medium eagle, Mp, metalloproteinase domain, OPN, osteopontin, PAH, pulmonary arterial hypertension, PA-SMC, pulmonary artery–smooth muscle cell, PEI, polyethylenimine, Pro, prodomain, Sp, spacer, TIMP, tissue inhibitor of metalloproteinase, TSR, thrombospondin-like motif, WT, wild type

## Abstract

A disintegrin-like and metalloprotease domain with thrombospondin type 1 motifs (ADAMTS)8 is a secreted protease, which was recently implicated in pathogenesis of pulmonary arterial hypertension (PAH). However, the substrate repertoire of ADAMTS8 and regulation of its activity are incompletely understood. Although considered a proteoglycanase because of high sequence similarity and close phylogenetic relationship to the proteoglycan-degrading proteases ADAMTS1, 4, 5, and 15, as well as tight genetic linkage with ADAMTS15 on human chromosome 11, its aggrecanase activity was reportedly weak. Several post-translational factors are known to regulate ADAMTS proteases such as autolysis, inhibition by endogenous inhibitors, and receptor-mediated endocytosis, but their impacts on ADAMTS8 are unknown. Here, we show that ADAMTS8 undergoes autolysis at six different sites within its spacer domain. We also found that in contrast to ADAMTS4 and 5, ADAMTS8 levels were not regulated through low-density lipoprotein receptor-related protein 1 (LRP1)-mediated endocytosis. Additionally, ADAMTS8 lacked significant activity against the proteoglycans aggrecan, versican, and biglycan. Instead, we found that ADAMTS8 cleaved osteopontin, a phosphoprotein whose expression is upregulated in PAH. Multiple ADAMTS8 cleavage sites were identified using liquid chromatography–tandem mass spectrometry. Osteopontin cleavage by ADAMTS8 was efficiently inhibited by TIMP-3, an endogenous inhibitor of ADAMTS1, 4, and 5, as well as by TIMP-2, which has no previously reported inhibitory activity against other ADAMTS proteases. These differences in post-translational regulation and substrate repertoire differentiate ADAMTS8 from other family members and may help to elucidate its role in PAH.

The ADAMTS (a disintegrin-like and metalloproteinase domain with thrombospondin motifs) family comprises 19 secreted proteases, with the first member described more than 20 years ago (1). ADAMTS proteases are involved in diverse biological processes and are implicated in various acquired diseases such as osteoarthritis and atherosclerosis, numerous birth defects and genetic disorders, such as Ehlers–Danlos syndrome (dermatosparactic type), Weill–Marchesani syndrome, and thrombotic thrombocytopenic purpura (2, 3). However, the functions of some ADAMTS proteases remain poorly understood, despite considerable recent research that has expanded the substrate repertoire of these enzymes, as well as their known biological roles (4). ADAMTS8, previously referred to as METH-2, is one such “orphan protease” that is predominantly expressed in the lung and heart (5, 6, 7) and is reported to be downregulated *via* promoter hypermethylation in a variety of cancers (8, 9, 10, 11, 12, 13). Reduced expression of ADAMTS8 is frequently associated with cancer cell invasion and metastasis (8, 9, 12, 14, 15).

Recently, ADAMTS8 expression was found to be increased in lungs of patients with pulmonary arterial hypertension (PAH) (7). In addition, mice bearing a targeted deletion of *Adamts8,* either in their pulmonary artery smooth muscle cells (PA-SMCs) or cardiomyocytes, showed reduced right ventricular systolic pressure and reduced right ventricular hypertrophy, compared with wild-type (WT) mice when subjected to hypoxia-induced PAH. These results suggested a role for ADAMTS8 in the pathogenesis of PAH, potentially contributing to the severity of this condition (7). However, the biological roles of ADAMTS8 and its proteolytic activity are still poorly understood due to lack of biochemical characterization and elucidation of its substrate repertoire.

ADAMTS8 comprises a prodomain (Pro), a metalloproteinase (Mp) domain, a disintegrin-like (Dis) domain, a central thrombospondin-like (TSR) motif, a cysteine-rich (CR) domain, a spacer (Sp) domain, and a C-terminal TSR motif (Fig. 1*A*). ADAMTS8 has the highest sequence homology with ADAMTS1, 4, 5, and 15, four ADAMTS family members endowed with the distinct ability to cleave extracellular matrix (ECM) proteoglycans (4), and phylogenetic analysis has placed it in the evolutionary cluster that contains these proteases (16). The *ADAMTS8* gene locus is tightly linked with the *ADAMTS15* locus in both humans and mice (PMID17509843), suggesting an especially close relationship with this protease, about which there is also scarce information. Although ADAMTS8 is therefore considered a proteoglycanase, its only reported substrate is aggrecan, the predominant proteoglycan in articular cartilage (17). However, aggrecan was cleaved by ADAMTS8 only at extremely high enzyme/substrate ratios, suggesting that it may not be a major or preferred substrate (17). The ability of ADAMTS8 to cleave other proteoglycans, such as versican and biglycan, has so far not been reported. Post-translationally, ADAMTS proteases are regulated by tissue inhibitors of metalloproteinases (TIMPs), interactions with cell-associated glycosaminoglycans, and endocytic clearance by low-density lipoprotein receptor-related protein 1 (LRP1) (18). Whether ADAMTS8 is regulated through these mechanisms has not yet been investigated but may have an immediate relevance for its activity in diseases such as PAH.

**Figure 1:**
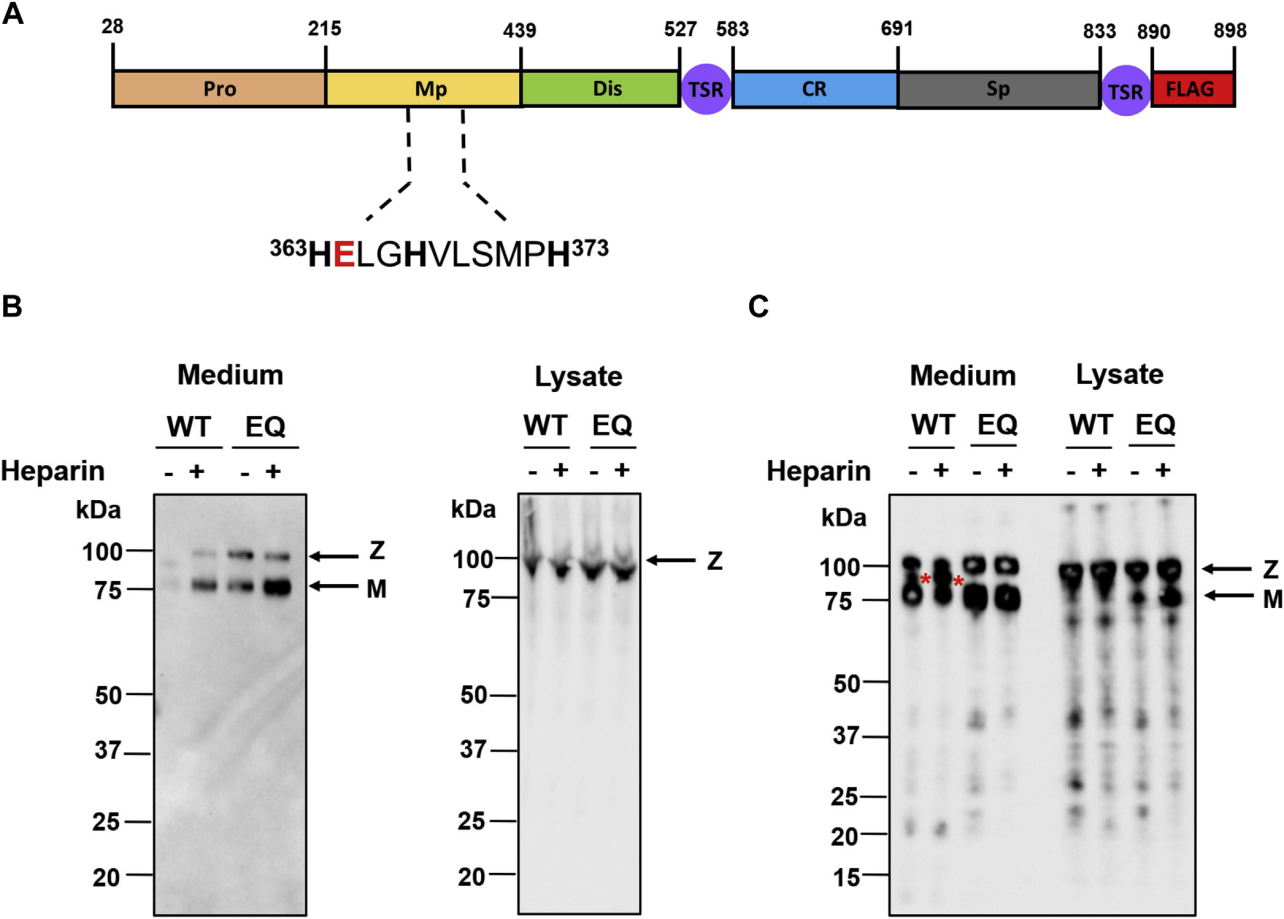
**Expression of recombinant ADAMTS8.***A*, annotated ADAMTS8 domain structure. The zinc-binding sequence (residues 363–373) is shown below the cartoon. Histidine residues involved in zinc coordination are in *bold*, the active site glutamic acid residue is in *red*. *B*, expression of recombinant ADAMTS8 in HEK293T cells. Cells were transiently transfected using PEI. 4 h post-transfection, the cells were treated with either heparin (200 μg/ml)-containing medium or an equal volume of expression medium. Three days post-transfection, the conditioned medium was harvested and protein lysate extracted as reported in the Experimental procedures. Samples were analyzed by 4–12% reducing SDS-PAGE, followed by immunoblotting using an anti-FLAG antibody. *C*, immunoblot as in (*B*) but with approximately 4-fold higher protein content/lane to highlight additional bands. *Red asterisks* indicate major autolytic products. *Z* and *M* indicate the zymogen and mature metalloproteinase, respectively. Representative blots from n = 3 are presented. CR, cysteine-rich domain; Dis, disintegrin-like domain; Mp, metalloproteinase domain; Pro, prodomain; Sp, spacer domain; TSR, thrombospondin-like motif.

To fill the gap in our understanding of ADAMTS8 as a protease and potential proteoglycanase, we generated purified recombinant ADAMTS8 and characterized its proteolytic activity, susceptibility to TIMP inhibition and regulation by LRP1-mediated endocytosis. We report here that ADAMTS8 exhibits striking differences in substrate repertoire, susceptibility to endogenous inhibitors, and endocytic regulation compared with ADAMTS1, 4, 5, and 15. Our results therefore suggest that ADAMTS8 is functionally distinct from other members of the proteoglycanase subfamily, despite its homology and evolutionary relationship with that group.

## Results

### Purification of recombinant ADAMTS8 and its active site mutant

Constructs expressing FLAG-tagged full-length WT ADAMTS8, and its active site mutant (E364Q, hereafter designated as “EQ”), were transiently expressed in HEK293T cells, an expression system previously used successfully for purification of recombinant ADAMTS1, 4 and 5 (19). Since ADAMTS proteases are known to bind ECM *via* ionic interactions, frequently requiring heparin for release into the culture medium (1, 20), heparin was added 4 h post-transfection to test its effect on ADAMTS8 levels. While heparin had little effect on the ADAMTS8 levels in the cell lysate, increased levels of ADAMTS8 (both WT and EQ) were present in the conditioned medium when heparin was added during expression (Fig. 1*B*). These results suggest that ADAMTS8 remains bound to the cell-ECM monolayer unless heparin is added. Once released into the medium, ADAMTS8 was present in two forms, with apparent molecular weights of ∼100 and 75 kDa, corresponding to the predicted size of zymogen and furin-cleaved mature (activated) forms of the enzyme, respectively. Addition of heparin mainly increased the abundance of the furin-activated form of ADAMTS8 and ADAMTS8 EQ (Fig. 1*B*). While WT ADAMTS8 was detected at much lower levels than ADAMTS8 EQ in the medium, both in the presence and in the absence of heparin, similar levels of the two forms were present in the cell lysate regardless of whether heparin was added or not (Fig. 1, *B* and *C*). Immunoblots with higher protein content loaded on the gel showed the presence of additional bands in both WT and EQ transfections in conditioned media and cell lysates, but bands intermediate in size between those corresponding to the zymogen and the mature forms of the enzyme were present exclusively in the WT transfections (Fig. 1*C*). Together, the results suggest that WT ADAMTS8 may undergo autolysis in the extracellular milieu, likely following furin activation, similar to that previously shown for other ADAMTS proteases (21, 22).

**Figure 2:**
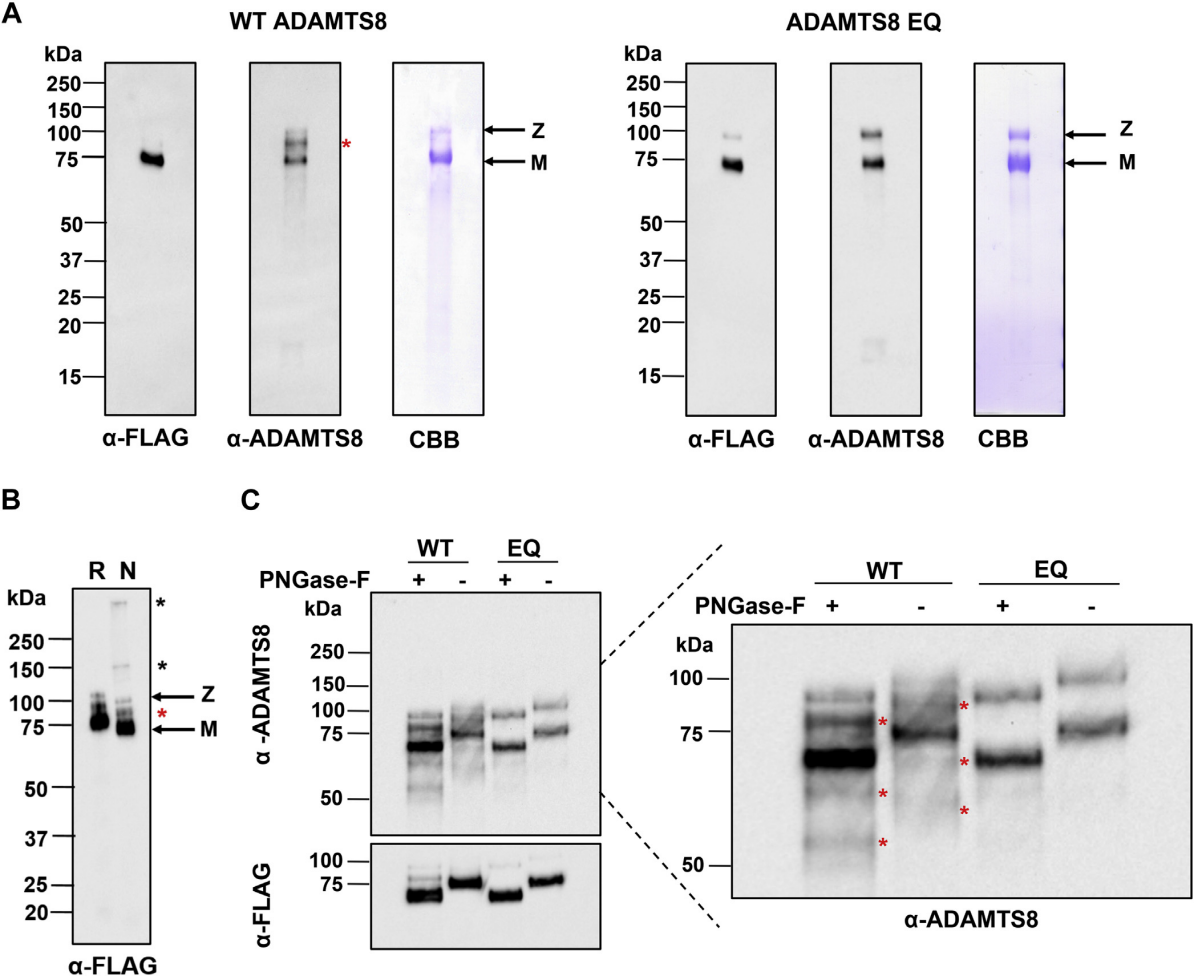
**Characterization of purified recombinant ADAMTS8.***A*, characterization of purified WT ADAMTS8 and ADAMTS8 EQ. The purified proteins were analyzed by reducing SDS-PAGE, followed by immunoblotting using either anti-FLAG or anti-ADAMTS8 antibodies. Protein purity was assessed by Coomassie Brilliant Blue (CBB). *B*, ADAMTS8 WT was subjected to SDS-PAGE under reducing (R) or non-reducing (N) conditions, followed by immunoblotting using anti-FLAG antibody. *C*, to detect N-linked glycosylation in ADAMTS8, WT and EQ ADAMTS8 were treated with (+) or without (−) PNGase before reducing SDS-PAGE (4–12%) and immunoblotting. *Red asterisks* indicate potential autolytic products; *black asterisks* indicate possible aggregates of ADAMTS8. *Z* and *M* indicate the zymogen and mature forms, respectively. Representative blots from at least n = 3 are presented.

Both ADAMTS8 constructs were subsequently expressed at larger scale in the presence of heparin and purified using anti-FLAG affinity chromatography. Since heparin binds strongly to ADAMTS proteases and has the potential to inhibit their proteolytic activity (20), anti-FLAG-bound ADAMTS8 was washed with high concentrations of NaCl (1 M) to ensure heparin removal, as previously reported (19, 20, 23). Following competitive elution with FLAG peptide, the two ADAMTS8 constructs were subjected to buffer exchange to remove the FLAG peptide and quantified by absorbance as described in the Experimental procedures section. The total yields of WT and EQ ADAMTS8 were 1.8 and 4.4. mg/l, respectively, approximately 60- and 36-fold higher than similarly purified WT ADAMTS5 (30 μg/l) (21) and ADAMTS4 (50 μg/l), respectively (20). The molecular forms and their purity were assessed using immunoblot and Coomassie Brilliant Blue staining (Fig. 2*A*). Both anti-FLAG and polyclonal anti-ADAMTS8 antibodies detected the zymogen as well as mature forms in each preparation. Both antibodies also detected additional bands, which were present only in WT ADAMTS8 before as well as after purification (Figs. 2, *A* and *B* and 1*C*), suggesting autolysis. To further verify protein quality, the WT ADAMTS8 preparation was subjected to SDS-PAGE under reducing and non-reducing conditions (Fig. 2*B*). More rapid migration under non-reducing conditions was indicative of correctly formed intramolecular disulfide bonds and a native-like conformation. Under reducing conditions, both the zymogen and mature forms migrated through the gel at a slightly higher molecular weight than predicted by their amino acid sequences (approximately 100 and 75 kDa *versus* 95 and 70 kDa, respectively). This suggested the presence of a post-translational modification such as glycosylation in the secreted protein. NetNGlyc 1.0 Server (http://www.cbs.dtu.dk/services/NetNGlyc/) predicted five potential N-glycosylation sites in ADAMTS8: one in the Pro domain (^344^N), one in the Mp domain (^400^N), two in the Dis domain (^465^N and ^490^N), and one in the CysR domain (^599^N). To verify the presence of N-linked glycosylations, purified ADAMTS8 WT and EQ were treated with PNGase-F prior to SDS-PAGE and immunoblot analysis (Fig. 2*C*). A shift in molecular weight of ∼5 kDa was observed between PNGase F-treated and untreated conditions, resulting in observed protein masses similar to those predicted for the two proteins, suggesting that the higher than expected molecular mass resulted from addition of N-linked glycans. PNGase-F treatment also highlighted several additional bands, which, like those observed in Figures 2, *A* and *B* and 1*C*, were specifically seen in WT ADAMTS8. We hypothesized that these bands may represent glycoforms of autolytic products.

### Determination of autolytic cleavage sites in ADAMTS8

**Figure 3:**
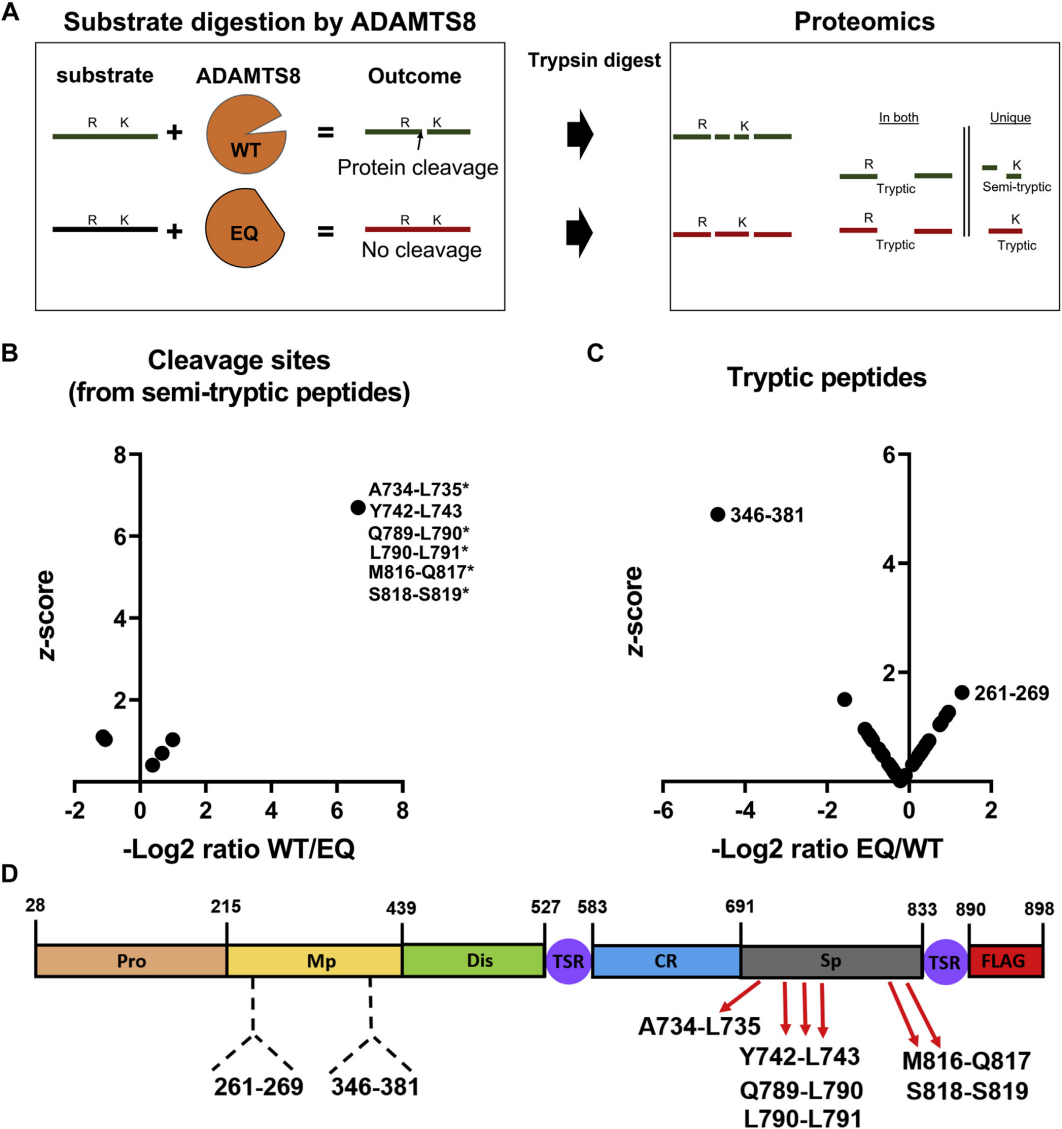
**ADAMTS autocatalysis.***A*, schematic showing how ADAMTS8 WT and EQ semi-tryptic and/or tryptic peptides with differential abundance were used to identify putative autolytic cleavage sites. Digestion of a general substrate is shown. This can be either a different protein or, as presented in (*B*–*D*), ADAMTS8 itself to define autolytic products. *B*, all identified N-terminal or C-terminal (∗) semi-tryptic peptide abundance was compared to obtain a ratio (WT/EQ) and z-score. Cleavage sites are identified by amino acid and residue number with the hyphen indicating the scissile bond. *C*, all tryptic peptides were compared by quantified ratio (EQ/WT) and z-score. For both (*A* and *B*) a z-score >2 was considered significant (specifics can be found in Table S1). *D*, ADAMTS8 domain map showing the location of the putative cleavage sites (*red arrows*) and tryptic peptides (*dashed black brackets*).

Since autolysis could remove domains necessary for proteolytic activity, we tested our hypothesis that ADAMTS8 undergoes autolysis using high-resolution liquid chromatography–tandem mass spectrometry (LC-MS/MS), applying a method we previously developed to identify novel cleavage sites in versican (24). We identified ADAMTS8 semi-tryptic and tryptic peptides and determined their relative abundance in medium from WT and EQ constructs with this label-free quantitative approach. Identifying peptides with a semi-tryptic N-terminus (indicating the P1′ amino acid residue just downstream of the scissile bond, nomenclature after Schecter & Berger) (25) or a semi-tryptic C-terminus (*i.e.*, indicating the P1 amino acid residue) at quantitatively higher abundance in WT ADAMTS8 would suggest putative sites of autolysis. Furthermore, identification and quantitation of the corresponding tryptic peptides bridging/spanning the putative cleavage sites at higher abundance in ADAMTS8 EQ would provide additional support for the identified ADAMTS8 autolytic cleavages (Fig. 3*A*). Following a 24 h incubation of either WT or EQ ADAMTS8, seven semi-tryptic internal peptides were identified only in WT ADAMTS8 preparations (Figs. 3*B* and S1 and Table S1), including the N-terminal and C-terminal semi-tryptic peptides indicating a cleavage site at Ala^734^- Leu^735^. The analysis suggested six sites where WT ADAMTS8 was subject to autolysis: Ala^734^- Leu^735^, Tyr^742^-Leu^743^, Gln^789^-Leu^790^, Leu^790^-Leu^791^, Met^816^-Gln^817^, and Ser^818^-Ser^819^ (Fig. 3*B*). Interestingly, all of these cleavage sites are located within the ADAMTS8 Sp domain (Fig. 3*D* and Table S1), which in other ADAMTS proteases is involved in substrate recognition (19). Only a single tryptic peptide comprising residues 346 to 381 was significantly higher in the WT compared with the EQ (z-score of 4.9). This peptide may indicate a tryptic-like autolytic specificity of ADAMTS8 although it could conceivably result from a contaminant protease, which is an unlikely possibility since both constructs were expressed under identical conditions. No tryptic peptides were observed at significantly higher levels in the EQ as compared with the WT, with the highest being the peptide comprising residues 261 to 269 and having a z-score of only 1.6 (Fig. 3*C*).

### ADAMTS8 is not regulated by LRP1-mediated endocytosis

**Figure 4:**
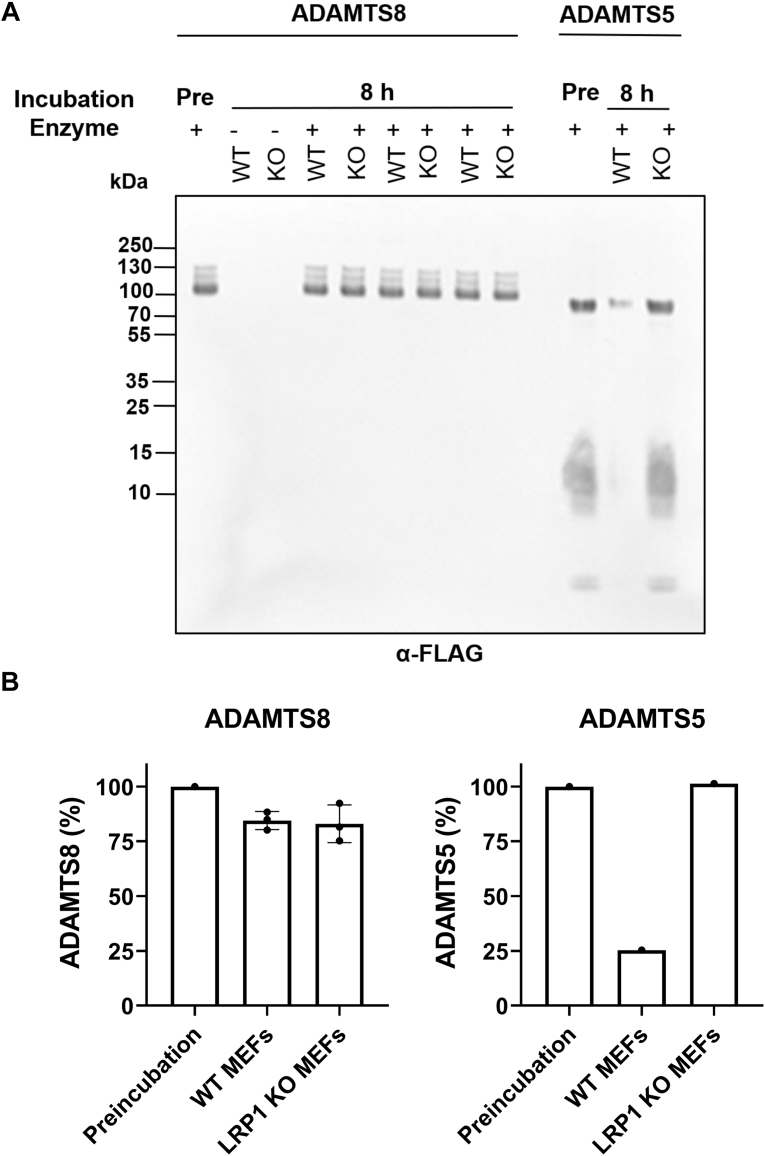
**Endocytosis of ADAMTS8 by LRP1 WT and LRP1-knockout MEFs.** WT and LRP1 knockout (KO) MEFs were cultured for 8 h in the presence (+) or absence (−) of either ADAMTS8 or ADAMTS5 (20 nM). Medium was harvested, trichloroacetic acid-precipitated, and resuspended in SDS sample buffer containing 5% mercaptoethanol. Following SDS-PAGE and immunoblotting, ADAMTS5 and 8 were detected by an anti-FLAG antibody. Band intensity was quantified by densitometry. Intensity of the bands corresponding to either ADAMTS8 or ADAMTS5 before incubation (pre) was set as 100%. *A*, immunoblot showing three biological replicates. *B*, densitometric analysis of FLAG-reactive bands. Data are presented as mean ± SD. In the case of ADAMTS5, only a single biological replicate is shown.

Heparin increased ADAMTS8 levels in the medium of HEK293T cells (Fig. 1*B*). Heparin is therefore likely to bind to ADAMTS8 and exert this effect by either competing with binding to ECM, competing with binding to LRP1 or a combination of both scenarios (26). LRP1 is known to post-translationally regulate the activity of ADAMTS4 and ADAMTS5 through their endocytic clearance from the extracellular milieu (27, 28). We aimed to establish if ADAMTS8 also undergoes such post-translational regulation. We found that ADAMTS8 did not bind purified human LRP1 in a solid-phase binding assay (Fig. S2), suggesting that it may not be internalized through this receptor. We therefore compared the uptake of WT ADAMTS8 by LRP1 WT mouse embryonic fibroblasts (MEFs) and LRP1 knockout MEFs. Purified recombinant WT ADAMTS8 was added to either LRP1 WT or LRP1 knockout MEFs. After 8 h incubation, media were collected, proteins were precipitated with trichloroacetic acid, dissolved in SDS-sample buffer, and analyzed by SDS-PAGE under reducing conditions (Fig. 4). No difference was observed between LRP1 WT and LRP1 knockout MEFs, with approximately 85% of the starting material present at the end of the incubation period. In comparison, approximately 75% of ADAMTS5 disappeared from the medium of WT LRP1 MEFs, while its levels were maintained in medium incubated with LRP1 knockout cells (Fig. 4). These results suggest that, in contrast to ADAMTS5, ADAMTS8 is not regulated by LRP1-mediated endocytosis.

### ADAMTS8 lacks appreciable proteolytic activity against aggrecan, biglycan, and versican

**Figure 5:**
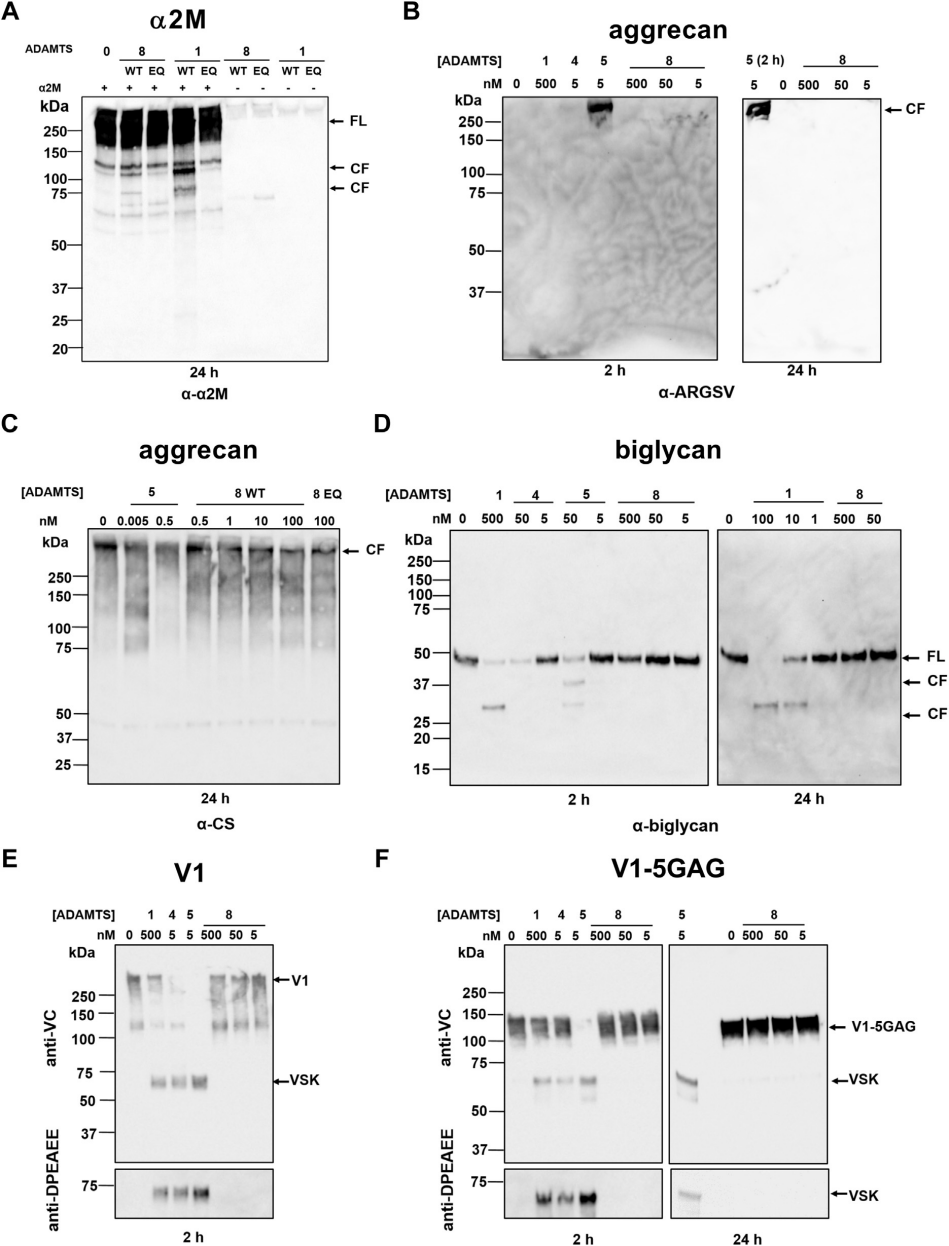
**Proteolytic activity of ADAMTS8.***A*, ADAMTS8 cleaves α2-macroglobulin. α_2_M (250 nM) was incubated with either ADAMTS8 or ADAMTS1 (250 nM) for 24 h. Samples were subjected to 4 to 12% SDS-PAGE and probed with polyclonal anti-α_2_M antibodies. Full-length (FL) substrates and specific cleavage fragments (CF) are indicated. All reactions were performed with the WT or EQ form of each enzyme. *B*, activity of ADAMTS1 (500 nM), ADAMTS4 (5 nM), ADAMTS5 (5 nM), and ADAMTS8 (5–500 nM) against bovine aggrecan (667 nM). Cleavage fragments generated after either 2 or 24 h digestion (as indicated) were detected using the anti-ARGSV neoepitope antibody BC3. *C*, activity of ADAMTS5 (0.005–0.5 nM) and ADAMTS8 (0.5–100 nM) against aggrecan (667 nM) after 24 h digestion. Activity of 100 nM ADAMTS8 EQ is shown for comparison. Bands were detected by the antibody 2B6, recognizing the CS stubs remaining following treatment of aggrecan with chondroitinase ABC. *D*, activity of ADAMTS1 (1–500 nM), ADAMTS4 (5–50 nM), ADAMTS5 (5–50 nM), and ADAMTS8 (5–500 nM) against biglycan (2 μM) was investigated after 2 or 24 h digestion (as indicated) using polyclonal antibodies against biglycan. Full-length (FL) substrates and specific cleavage fragments (CF) are indicated. *E* and *F*, activity of ADAMTS1 (500 nM), ADAMTS4 (5 nM), ADAMTS5 (5 nM), and ADAMTS8 (5–500 nM) against versican V1 (*E*) and V1-5GAG (*F*) (each at 100 nM) after 2 and 24 h digestions. Bands were detected using anti-VC and anti-DPEAEE antibodies. All digestions were performed at 37 °C. Representative blots from n = 3 independent replicates are presented. VSK, versikine.

Following overall protein characterization, the enzymatic activity of purified recombinant ADAMTS8 was tested against a range of protein substrates. ADAMTS8 activity was initially tested against α2-macroglobulin (α2M), a general substrate and plasma inhibitor of metalloproteinases (Fig. 5*A*). Cleavage of α2M by proteases generally occurs in a bait region of ∼30 amino acids (^690^Pro-Thr^728^) (29). Following 24 h incubation at equimolar concentrations, ADAMTS8-mediated proteolysis of α2M was observed, confirming its enzymatic activity. The cleavage products were similar to those of ADAMTS1, here used as a positive control (Fig. 5*A*). Since ADAMTS8 was reported to cleave aggrecan (17), its aggrecanase activity was tested against purified bovine aggrecan and compared with that of ADAMTS1, ADAMTS4, and ADAMTS5, which cleave aggrecan at the Glu^392^-Ala^393^ bond (human aggrecan numbering, Uniprot accession number: P16112) (Fig. 5*B*). In the presence of as little as 5 nM ADAMTS5, a 250-kDa band, corresponding to the C-terminal cleavage fragment ^393^ARGSVIL, was detected by the neoepitope antibody BC3 after 2 h digestion. Only a faint band was detected in the ADAMTS4 digest (5 nM), in agreement with its lower aggrecanase activity compared with ADAMTS5 (20, 21), whereas neither ADAMTS1 nor ADAMTS8 was able to significantly cleave aggrecan even at concentrations up to 500 nM. Similar results were observed for ADAMTS8 following 24 h incubation with bovine aggrecan (Fig. 5*B*). To exclude the possibility that ADAMTS8 may cleave aggrecan at other sites, similar reactions were analyzed by immunoblot with 2B6, an antibody that recognizes the chondroitinase ABC-generated chondroitin 4-sulfate stubs (CS) remaining on chondroitin-sulfate proteoglycan core proteins after deglycosylation (30) (Fig. 5*C*). In the presence of 0.5 nM ADAMTS5, the intensity of the band corresponding to full-length aggrecan was decreased, demonstrating further processing at a variety of cleavage sites, as previously observed (21, 31). However, no such difference was observed upon incubation with up to 100 nM ADAMTS8 for 24 h, confirming the lack of significant aggrecanase activity.

Since ADAMTS8 is considered to be part of the proteoglycanase family of ADAMTS proteases, we tested its proteolytic activity against two other chondroitin-sulfate proteoglycans, biglycan and versican. Biglycan is a small leucine-rich proteoglycan, which can be cleaved by both ADAMTS4 and 5 (Fig. 5*D*) (21). Accordingly, ADAMTS4 and 5 cleaved biglycan at multiple sites with comparable efficiency, while ADAMTS1 had approximately 10-fold lower proteolytic activity (*i.e.*, the activity of 500 nM ADAMTS1 was comparable to that of 50 nM ADAMTS4 and ADAMTS5). To the best of our knowledge, this is the first report of ADAMTS1 activity against biglycan. In comparison, ADAMTS8 did not appreciably cleave biglycan even upon 24 h incubation at 500 nM. Finally, we tested ADAMTS8 activity against full-length versican V1 and a truncated construct, V1-5GAG (comprising amino acids 21–694) (32) (Fig. 5, *E* and *F*), which is cleaved as efficiently as V1 by ADAMTS1, ADAMTS4, and ADAMTS5 (19). After deglycosylation, cleavage products were analyzed by SDS-PAGE and immunoblot using anti-VC and anti-DPEAEE antibodies. Cleavage by ADAMTS1, ADAMTS4, and ADAMTS5 occurs at the Glu^441^-Ala^442^ site, generating versikine, an N-terminal cleavage product recognized by anti-DPEAEE neoepitope antibody (19, 32). The anti-VC antibody targets the sequence ^432^VPKDPEAAEARRG^445^ and can recognize full-length V1, V1-5GAG and versikine. We confirmed that ADAMTS5 was the most potent against the two versican constructs, followed by ADAMTS4 and ADAMTS1, as published previously (19). In contrast, ADAMTS8 did not show any proteolytic activity in these assays. Overall, these data suggest that ADAMTS8 lacks appreciable activity against these selected proteoglycans at these specific sites.

### ADAMTS8 cleaves the matricellular protein osteopontin

**Figure 6:**
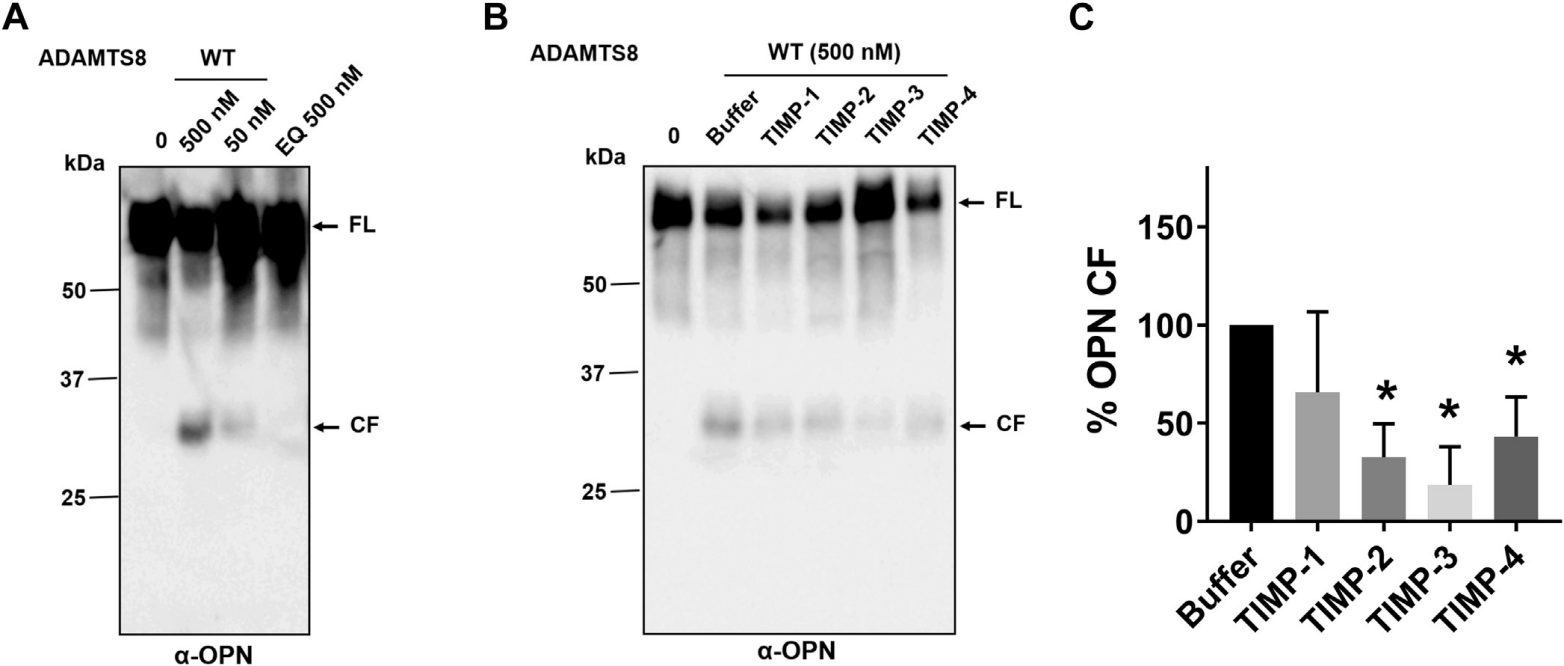
**ADAMTS8 cleaves human OPN.***A*, OPN (760 nM) was incubated with either ADAMTS8 WT or EQ (50–500 nM) for 24 h at 37 °C. Samples were subjected to 4 to 12% SDS-PAGE/immunoblot under reducing conditions and probed with a polyclonal anti-OPN antibody. *B*, inhibition of ADAMTS8 proteolytic activity. TIMP-1, -2, -3, and -4 (each at 1 μM) were incubated with ADAMTS8 (500 nM) for 1 h at 37 °C before addition of OPN. Following SDS-PAGE and immunoblotting, OPN fragments were detected by a polyclonal anti-OPN antibody. A representative immunoblot is shown (n = 4). Full-length (FL) substrates and specific cleavage fragments (CF) are indicated. *C*, densitometric analysis showing quantification of ADAMTS8-generated cleavage fragments (CF). Data are presented as mean ± SEM (n = 4). ∗*p* < 0.05 relative to buffer control, without inhibitor (Mann–Whitney test).

Since ADAMTS8 is implicated in PAH (7), we searched through the literature and the Gene Expression Omnibus (GSO) database for ECM proteins differentially expressed in PAH for identification of potential ADAMTS8 candidate substrates. The gene found to be most consistently upregulated was *Spp1*, coding for secreted phosphoprotein 1 or osteopontin (OPN) (Table S2). OPN stimulates growth and migration of PAH SMCs (33, 34) and contributes to the highly proliferative, migratory and proinvasive phenotype exhibited by adventitial fibroblasts in PAH (35). OPN plasma levels increase in patients with PAH (33, 35, 36, 37) and correlate with disease severity (37) and mortality (36, 38). OPN was therefore selected as a candidate ADAMTS8 substrate. Following incubation with purified WT ADAMTS8 (24 h), a prominent ∼32 kDa band was detected already at low ADAMTS8 concentrations (50 nM; enzyme/substrate ratio 0.1) and was more prominent at higher concentrations (500 nM; enzyme/substrate ratio 1) (Fig. 6*A*). Importantly, the same OPN species was absent in the presence of ADAMTS8 EQ, thus demonstrating the specificity of proteolysis. We also investigated whether OPN proteolysis could be inhibited by TIMPs, the endogenous inhibitors of ADAMTS proteases. Following SDS-PAGE and immunoblotting (Fig. 6*B*), inhibition of proteolysis was quantified by densitometric analysis of bands corresponding to ADAMTS8-cleaved OPN (Fig. 6*C*). TIMP-3 was the most potent inhibitor of ADAMTS8 activity, followed by TIMP-2, whereas TIMP-1 and TIMP-4 showed modest inhibition.

**Figure 7:**
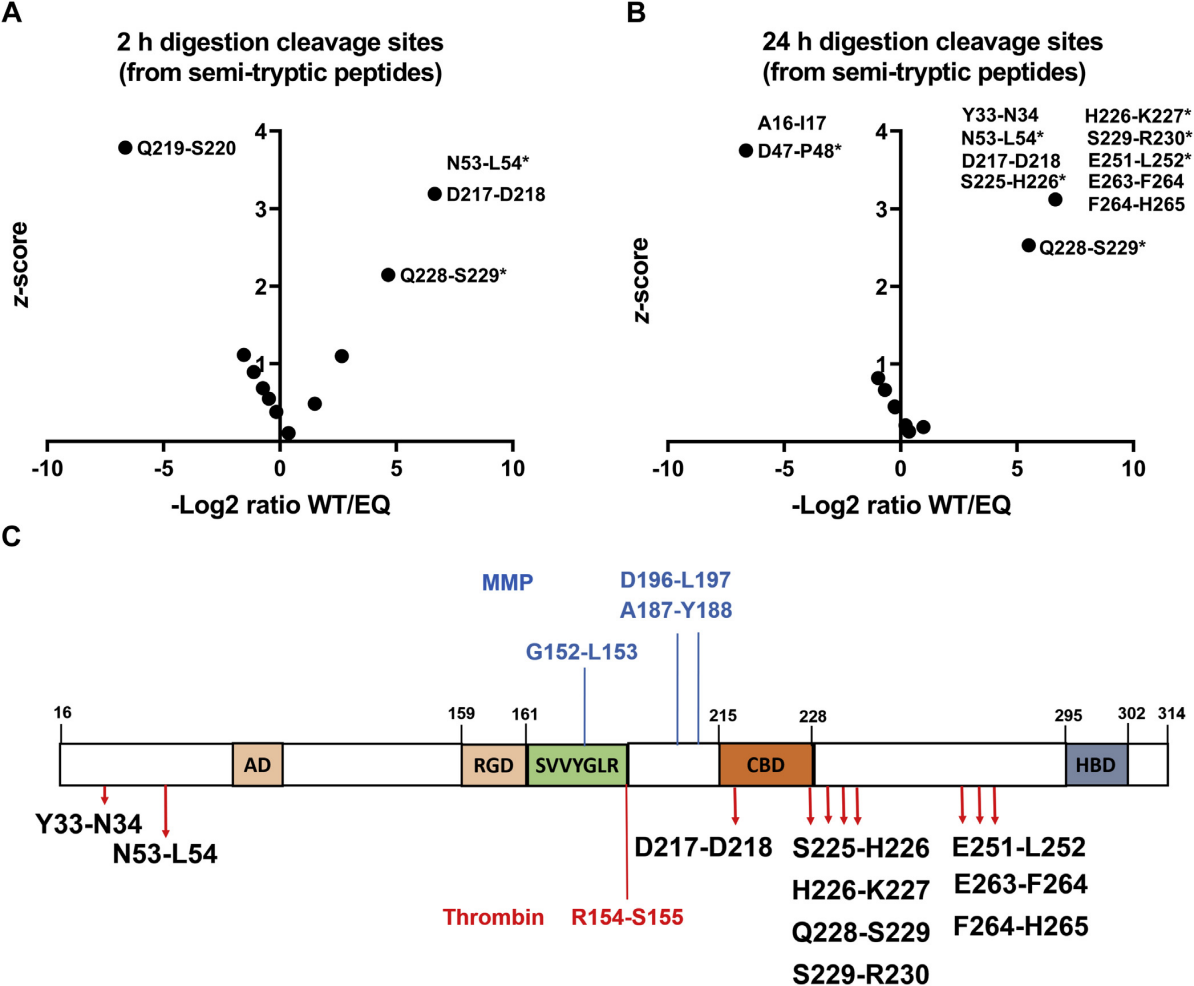
**Mapping OPN cleavage sites by ADAMTS8 using LC-MS/MS.***A* and *B*, semi-tryptic peptides detected in 2 h (*A*) or 24 h (*B*) digestions. All identified N-terminal or C-terminal (∗) semi-tryptic peptides were compared to obtain a ratio (WT/EQ) and z-score. Cleavage sites are identified by amino acid and residue number with the hyphen indicating the scissile bond (specifics can be found in Table S3 and Figs. S3–S5). *C*, the domain structure of OPN isoform b showing the location of the identified ADAMTS8 cleavage sites. Previously described cleavage sites of MMPs and thrombin are indicated in *blue* and *red*, respectively. AD, aspartate domain; CBD, calcium-binding site; HBD, heparin-binding site; RGD, arginine–glycine–aspartic acid sequence.

To identify the cleavage sites, OPN digests were analyzed using LC-MS/MS to identify semi-tryptic and tryptic peptides and determine their relative abundance in a label-free quantitative approach, similar to that used to identify ADAMTS8 autolysis products (Table S3). In the 2 h digests, four semi-tryptic internal peptides were significantly more abundant in WT ADAMTS8 digests, compared with the EQ control (Figs. 7*A* and S3). These included three C-terminal semi-tryptic peptides derived from proteolysis at cleavage sites Asn^53^-Leu^54^ and Gln^228^-Ser^229^ and one N-terminal semi-tryptic peptide derived from proteolysis at Asp^217^-Asp^218^(Fig. 7*A*). The peptide indicating cleavage at Asn^53^-Leu^54^ was identified as both a non-modified peptide and with a modified glutamine residue cyclized into pyroglutamate on the N-terminus. The peptide indicating cleavage at Gln^228^-Ser^229^ was 25-fold (log2 ratio of 4.6) higher in the ADAMTS8 digest compared with the the EQ control and had a z-score of 2.1. The other three peptides were identified only in the ADAMTS8 digest and were assigned a scaled fold-change of 100 (Table S3) giving them z-scores of 3.2. The 2 h digests produced no tryptic peptides with significant abundance differences between the ADAMTS8 and EQ control digest (Fig. S4*A*).

In the 24 h digestions (Figs. 7*B* and S5), the same four cleavage sites were identified as well as seven additional ones. The peptide indicating cleavage at Gln^228^-Ser^229^ was again significantly more abundant in the ADAMTS8 digest compared with the EQ control with both the fold-change and z-score increased relative to that of the 2 h digestion (fold change of 45, log2 ratio of 5.5, and z-score of 2.5) (Table S3). The same two peptides (one modified and one nonmodified) indicating cleavage at Asn^53^-Leu^54^ and the peptide indicating cleavage at Asp^217^-Asp^218^ were again only found in the ADAMTS8 digest. Additionally, seven new putative cleavage sites (from eight peptides) that were not identified in the 2 h digest were identified in the ADAMTS8 24 h digest (Fig. 7*B*). These included semi-tryptic C-terminal peptides indicating proteolysis at Tyr^33^-Asn^34^, Ser^225^-His^226^, His^226^-Lys^227^, Ser^229^-Arg^230^, and Glu^251^-Leu^252^, and the N-terminal semi-tryptic peptides indicating proteolysis at Glu^263^-Phe^264^, and Phe^264^-His^265^. Two peptides indicating proteolysis at Phe^264^-His^265^ were found, one un-modified and another with an oxidized methionine residue at position 270. No tryptic peptides showed significant abundance changes in the 24 h digestion (Fig. S4*B*). These results suggest that Tyr^33^-Asn^34^, Asn^53^-Leu^54^, Asp^217^-Asp^218^, Ser^225^-His^226^, His^226^-Lys^227^, Gln^228^-Ser^229^, Ser^229^-Arg^230^, Glu^251^-Leu^252^, Glu^263^-Phe^264^, and Phe^264^-His^265^ are likely sites of ADAMTS8 cleavage of OPN (Fig. 7*C* and Table S3).

Semi-tryptic peptides, which were found only in the EQ digestion and not in the WT ADAMTS8 digestion, were also identified in both the 2 h (one peptide) and the 24 h (two peptides) digestions (Fig. 7, *A* and *B*). As for those observed in Figure 3*C*, these peptides could be products of either a contaminating trypsin-like protease or semi-tryptic peptides, which span an area with a putative ADAMTS8 cleavage site. This was the case for the peptide spanning residues 31 to 47 (Table S3 and D^47^_P^48^ in Fig. 7*B*), spanning the cleavage site Y^33^-N^34^.

## Discussion

In contrast to many members of the ADAMTS family, ADAMTS8 is still poorly characterized. While a potential aggrecanase activity was suggested (17), no additional substrates are known. Here, we provide several important insights into ADAMTS8 biochemistry, showing that it is an N-glycosylated protease that binds to the ECM *via* ionic interactions that are disrupted by heparin and is cleaved autolytically upon secretion. In contrast to related ADAMTS proteases, we show that ADAMTS8 is not regulated by LRP1-mediated endocytosis. Moreover, we have undertaken a detailed analysis of a candidate substrate, OPN, and identified specific cleavages using LC-MS/MS, which were orthogonally validated by immunoblot.

ADAMTS8 has high sequence homology to the proteoglycanases ADAMTS1, ADAMTS4, and ADAMTS 5 (4). In fact, the domain organization of ADAMTS8 is the same as that of ADAMTS5 (approximately 50% sequence identity by Clustal Omega analysis: https://www.ebi.ac.uk/Tools/msa/clustalo/). Using a mammalian expression system previously used for ADAMTS1, ADAMTS4, and ADAMTS5 (19), we expressed and purified recombinant human ADAMTS8 WT and its EQ mutant. Once secreted, ADAMTS8 binds to the ECM, where it can be released by heparin, which likely acts by disrupting ionic interactions with ECM and cell-surface components such as glycosaminoglycans (5), as was shown previously for other ADAMTS family members, such as ADAMTS1 (1), ADAMTS4 (20), and ADAMTS5 (21). Analysis of the ADAMTS8 amino acid sequence highlighted a stretch of positively charged residues in the central TSR domain (^567^GRRAKY^572^), which corresponds to a consensus sequence for heparin binding (“XBBXBX”, where B indicates a basic residue and X any other amino acid) (39). Several metalloproteinases bind both heparin and LRP1, and they can compete with each other for binding to these ligands (25); however, ADAMTS8 lacks LRP1-binding ability, which may prolong ADAMTS8 activity in the extracellular milieu. Indeed, much higher levels of ADAMTS8 were detected in the medium of HEK293T cells, known to express LRP1, compared with ADAMTS4 and ADAMTS5 (40).

**Figure 8:**
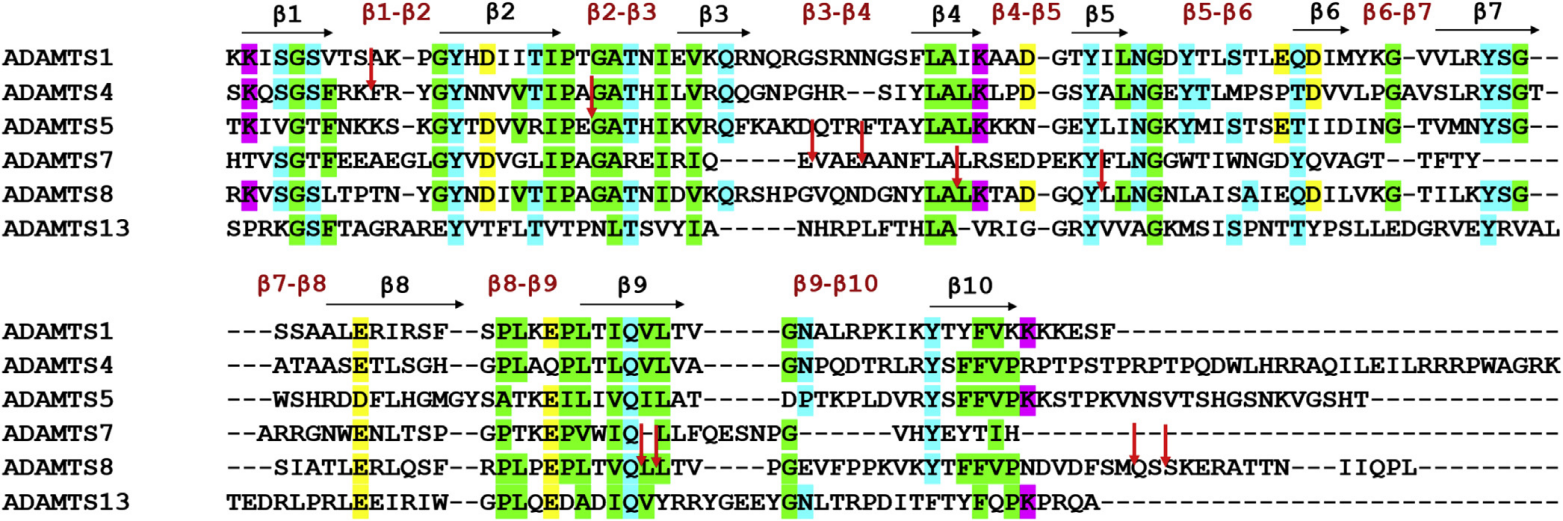
**Autolytic sites in ADAMTS spacer domains.** Amino acid sequence alignment (Clustal omega) of the Sp domain of human ADAMTS1 (Uniprot accession number: Q9UHI8, aa 725–749), ADAMTS4 (O75173, aa 686–837), ADAMTS5 (Q9UNA0, aa 732–874), ADAMTS7 (Q9UKP4, aa 698–809) 8 (Q9UP79, aa 690–831), and ADAMTS13 (Q76LX8, aa 556–685). Sequence identity of ADAMTS1, 4, 5, 7, and 13 were 58.8, 40.3, 34.1, 34.3, and 21.01%, respectively, compared with ADAMTS8. Beta strands and loops are indicated above the alignments in *black* and *red*, respectively. Conserved amino acids are colored according to their physicochemical properties (*pink*, positively charged; *yellow*, negatively charged; *green*, apolar; *cyan*, polar). *Red arrows* indicate autolytic cleavage sites identified in the present study as well as those reported in the literature (41, 42, 43).

Proteolysis of α2M confirmed that purified ADAMTS8 retained proteolytic activity. Using immunoblotting and LC/MS analysis, we also demonstrated that furin-activated ADAMTS8 undergoes autolysis within its Sp domain. Autolysis has been reported for several other ADAMTS proteases, including ADAMTS4 (41), ADAMTS5 (42), ADAMTS7 (43), and ADAMTS17 (44) where it typically occurs in the Sp domain. To date, the only available structure of a Sp domain of an ADAMTS family member is that of ADAMTS13 (45, 46), consisting of ten β-strands in a jelly-roll topology, connected by nine loops. Sequence alignment of ADAMTS8 with other ADAMTS proteases, including ADAMTS13, showed that the autolytic sites occur within the β4 and β9 strands, as well as at the end of the domain (Fig. 8). This contrasts with ADAMTS4, ADAMTS5, and ADAMTS7, where most autolytic sites are within the Sp loops. Whether these autolytic events actually occur *in vivo* for ADAMTS8 and contribute to post-translational regulation requires further investigation since the present analysis did not examine ADAMTS8 from natural sources. It would also be relevant to investigate the possibility that these sites are susceptible to cleavages by other proteases and to investigate substrate recognition by truncated forms of ADAMTS8. Specifically, as shown first for ADAMTS1 (1) and subsequently characterized in detail for ADAMTS5 (19, 20, 21), substrate recognition and ECM binding are largely a function of the ancillary domains. Hence full-length ADAMTS8 and truncation within the Sp domain by autolysis may significantly impact its proteolytic specificity and efficiency.

Although ADAMTS8 was reported to cleave aggrecan at the Glu^392^-Ala^393^ bond, it did so at a minimal ADAMTS8/aggrecan ratio of 1:0.2 (17). We found that ADAMTS8 lacks any detectable aggrecanase activity at this site at an ADAMTS8/aggrecan ratio of 1:1.5. Further experiments using an anti-CS stub antibody also failed to detect appreciable cleavage at other sites in aggrecan. While it is possible that ADAMTS8 may cleave aggrecan at higher ADAMTS8/aggrecan molar ratios, our results strongly suggest that aggrecan is not a physiologically relevant substrate for ADAMTS8. Indeed, ADAMTS8 expression in cartilage is not significantly altered in murine models of osteoarthritis (47, 48) as would be expected if ADAMTS8 is critically involved in aggrecan turnover. In addition to aggrecan, ADAMTS8 did not cleave versican, a closely related large aggregating proteoglycan, or the small leucine-rich proteoglycan biglycan. Together, these data suggest that ADAMTS8, despite being homologous to ADAMTS1, ADAMTS4, and ADAMTS5, has a potentially distinct substrate repertoire.

Accordingly, we considered the possibility of other potential ADAMTS8 substrates. We selected OPN as a candidate substrate, since it is consistently upregulated in PAH, where ADAMTS8 has a mechanistic role (7). Although *Spp1*^−/−^ mice have yet to be tested in a PAH model, they notably exhibit reduced total lung capacity, increased lung compliance and alveolar size (49), suggesting that optimal OPN levels are essential for lung development and/or maintaining lung function. Furthermore, small interfering RNA (siRNA)-mediated *Spp1* knockdown mitigated lung fibrosis in a pulmonary fibrosis model (50). PAH patients show increased expression of OPN (33, 35, 36, 37), and prior studies have shown that both ECM-bound and serum OPN levels correlate with disease severity (37). Increased OPN levels are also an independent predictor of mortality (36) and of adverse right ventricular remodeling and dysfunction (51, 52) in PAH patients, irrespective of the underlying aetiology. Setting up digestion reactions using 50 or 500 nM ADAMTS8, we found that ADAMTS8 cleaved OPN at a minimal enzyme/substrate molar ratio of 0.07. OPN is a secreted adhesive phosphoglycoprotein containing multiple functional motifs and domains. From N-terminus to C-terminus, these include the arginine-glycine-aspartic acid (RGD) cell-binding sequence, a serine-valine-valine-tyrosine-glycine-leucine-arginine (SVVYGLR) domain, mediating the interaction between OPN and integrin receptors α4β1 and α9β1, a calcium-binding site, and a heparin-binding site. Our LC-MS analysis of OPN digests by ADAMTS8 identified cleavage sites at Tyr^33^-Asn^34^, Asn^53^-Leu^54^, Asp^217^-Asp^218^, Ser^225^-His^226^, His^226^-Lys^227^, Gln^228^-Ser^229^, Ser^229^-Arg^230^, Glu^251^-Leu^252^, Glu^263^-Phe^264^, and Phe^264^-His^265^. Three of these, Asn^53^-Leu^54^, Asp^217^-Asp^218^ and Gln^228^-Ser^229^, were significant already after 2 h digestion. Cleavage at Asp^217^-Asp^218^ could potentially disrupt the calcium-binding site, which mediates binding to the hyaluronan receptor CD44 (53). Furthermore, OPN peptides derived by cleavage at Tyr^33^-Asn^34^, Asn^53^-Leu^54^, Gln^228^-Ser^229^, Ser^225^-His^226^, Ser^229^-Arg^230^, Glu^251^-Leu^252^, Glu^263^-Phe^264^, were reported in human urine (54) and may represent diffusible products of ADAMTS8 or other protease activity on OPN. Future studies should address how the observed cleavages may affect the biological functions of OPN. Cleavage of OPN by matrix metalloproteinases (MMPs) occurs at Gly^152^-Leu^153^, Ala^187^-Tyr^188^, Asp^196^-Leu^197^ (54) (OPN isoform b numbering), which are distinct from those we attribute to ADAMTS8 proteolysis. These cleavage events have been shown to enhance the ability of the OPN RGD sequence to interact with CD44 as well as with ανβ1, ανβ3, and ανβ5 integrin receptors, thus increasing the pro-adhesiveness of OPN (55). Moreover, recombinant ADAMTS8 was shown to promote expression and secretion of MMP2, MMP9, MMP12, and MMP13 by PA-SMCs (7). It is therefore plausible that ADAMTS8 may regulate levels of full-length OPN both directly and indirectly, although this needs to be investigated in relevant mouse models of PAH. Although OPN cleavage by ADAMTS8 was inhibited most efficiently by TIMP-3, TIMP-2 also showed significant inhibition. For comparison, ADAMTS4 and 5 are inhibited exclusively by TIMP-3 (56), ADAMTS7 is most efficiently inhibited by TIMP-4 and modestly by TIMP-3 (43), whereas ADAMTS2 is inhibited by TIMP-3 (57), while neither ADAMTS13 nor ADAMTS15 is reported to be inhibited by TIMPs (58). TIMP-2 inhibits all MMPs with broad selectivity, as well as A Disintegrin-like and Metalloproteinase (ADAM)10 and 12 (59), but has not been previously reported as an ADAMTS inhibitor. Importantly, both TIMP-2 and TIMP-3 are expressed in PA-SMCs (60, 61, 62), which is of particular interest considering the association between ADAMTS8 and PAH.

A limitation of the current immunoblot-based assay to detect OPN cleavage is that is semi-quantitative at best and as such cannot provide inhibition constants. Moreover, relatively high enzyme concentrations and incubation times were required to obtain robust OPN cleavage. It is likely that there are other, undiscovered substrates that may be cleaved more efficiently by ADAMTS8. Therefore, these inhibition studies should be performed in the presence of substrates against which ADAMTS8 exhibits a higher catalytic efficiency (*k*_cat_/*K*_m_) than OPN. These novel substrates could emerge from agnostic degradomics techniques such as Terminal Amine Isotopic Labeling of Substrates (TAILS) (43). However, our results present the first evidence that TIMP-2 may function as an endogenous ADAMTS8 inhibitor.

In conclusion, our results shed new light on ADAMTS8 functionality, specifically its distinct substrate specificity and post-translational regulation. Rather than being a typical proteoglycanase, as its homology with ADAMTS1, ADAMTS4, ADAMTS5, and ADAMTS15 suggests, ADAMTS8 may have an entirely different substrate repertoire, which needs to be further investigated using unbiased approaches. The functional impact of its activity against OPN also represents an important line of investigation for the future, given the compelling significance of this matricellular protein for human disorders, especially PAH.

## Experimental procedures

### Expression and purification of recombinant ADAMTS8

A construct encoding for full-length WT human ADAMTS8 (Uniprot ID: Q9UP79) with an in-frame C-terminal FLAG tag (DYKDDDDK) was synthesized and cloned into pcDNA 3.1 (+) by Life Technologies. The ADAMTS8 EQ construct (containing the mutation Glu364→Gln) was generated by site-directed mutagenesis.

Human embryonic kidney cells expressing the SV40 large T antigen (HEK293T) were cultured in Minimum Essential Medium Eagle (MEM) (Sigma-Aldrich) with 10% fetal bovine serum (Labtech), 1 U/ml Penicillin and 0.1 mg/ml Streptomycin (Pen/Strep) (Sigma-Aldrich), 2 mM L-glutamine (Life Technologies), and 1× nonessential amino acids (Sigma-Aldrich) at 37 °C, with 5% CO_2_. Constructs were transiently transfected using polyethylenimine (PEI) (Polysciences Europe GmbH) (PEI/cDNA ratio: 3.6). Cells were washed with phosphate-buffered saline to remove fetal bovine serum, and the medium was replaced with Opti-MEM Gibco (Life Technologies) containing Pen/Strep (1 U/ml and 0.1 mg/ml, respectively) and 2 mM CaCl_2_. After 4 h, heparin from porcine intestinal mucosa (Sigma-Aldrich, Cat. no: H3393, 200 μg/ml) was added to release ADAMTS8 from the ECM. After the transfection, cells were incubated for 3 days before harvesting. Protein lysate was extracted using CelLytic MT Cell Lysis Reagent (Sigma-Aldrich Cat. no: C3228) according to the manufacturer’s instructions.

Conditioned medium (750 ml) was harvested and centrifuged for 20 min at 1500*g*, followed by filtration (0.45 μm) to remove cell debris and concentrated to 50 ml with the Tangential Flow Titration (Millipore) with a 30 kDa molecular weight cutoff. Purification was performed in batch mode using *Proteus* 1-step midi spin columns (Generon, Cat. n.: NB-45-00058-2). Medium was incubated with α-FLAG M2 Affinity Gel (Sigma-Aldrich, Cat. n: A2220), which was pre-equilibrated with 15 ml of TNC-B buffer (20 mM Tris/HCl pH 7.45, 150 mM NaCl, 10 mM CaCl_2_, 0.05% Brij-35), for 2 h at 4 °C. The resin was washed with TNC-B buffer containing 1 M NaCl to remove heparin (19, 20, 23) and bound proteins were eluted with 200 μg/ml FLAG peptide (Sigma-Aldrich, Cat. n.: F3290). The purified samples were then passed through a PD-10 column (GE Healthcare) pre-equilibrated in TNC-B buffer to remove the FLAG peptide. Aliquots taken from each purification step and the purified protein were analyzed using sodium dodecyl sulfate–polyacrylamide gel electrophoresis (SDS-PAGE), followed by immunoblot and Coomassie Brilliant Blue staining. Samples were analyzed under reducing (5% β-mercaptoethanol) and non-reducing conditions on 4 to 12% Bis-Tris NuPage Gels (Life Technologies). For immunoblotting, anti-ADAMTS8 sheep polyclonal antibody (Cat. no: AFF6614 R&D systems, Abingdon, UK 1 μg/ml, generated to residues 29–691 as immunogen) and mouse monoclonal anti-FLAG M2 antibody (Cat. no: F3165 Sigma Aldrich, 1 μg/ml) were used. Following addition of appropriate secondary horseradish peroxidase (HRP) antibodies (Agilent Technologies), Immobilon Chemiluminescent HRP substrate (Merck Millipore) was detected with a Chemidoc Touch Imaging system (Bio-Rad), and images were exported using Image lab software version 5.2.1 (Bio-Rad). Fractions containing pure ADAMTS8 were pooled, concentrated, and stored at −80 °C before activity assays. Protein concentration was measured using Nanodrop and calculated according to the Beer–Lambert law, using extinction coefficient 95,195 M^1^cm^−1^ and the Expasy ProtParam web tool.

### Expression and purification of ADAMTS1, 4, and 5 and versican variants

ADAMTS1, 4, and 5 were expressed, purified, and quantified as previously reported (19). Full-length versican V1 (32) was purified by anion exchange chromatography as previously described using HiTrap DEAE Sepharose (GE Healthcare) (19). Versican V1-5GAG, which is a truncated version of V1, comprising amino acids 21 to 694 of V1 with C-terminal C-myc/6× His tag was described previously (19, 32). V1-5GAG was purified according to previously established protocols using nickel affinity purification (19).

### Peptide N-glycosidase F (PNGase-F) digestion

ADAMTS8 (670 nM) was incubated either with PNGase-F (1 mU/μl, Sigma-Aldrich, Cat. n.: P7367) (16 h, 37 °C) or an equal amount of TNC-B buffer, before analysis by SDS-PAGE.

### Endocytosis assay

WT and LRP1 knockout MEFs were generated as described previously (63) and kindly provided by Professor Dudley Strickland (University of Maryland School of Medicine). Cells (1 × 10^4^/well) cultured in 96-well plates (precoated with 0.1% gelatin for 3 h) were rested in 100 μl of Dulbecco’s Modified Eagle’s Medium (DMEM) for 1 day. The medium was replaced with 50 μl of fresh DMEM containing either ADAMTS8 or ADAMTS5 (20 nM). After 8 h, 30 μl of medium was collected and mixed with 10 μl of four × SDS-sampling buffer containing 5% β-mercaptoethanol. All samples were analyzed by SDS-PAGE under reducing conditions and immunoblotting using anti-FLAG M2. Immune signals for exogenously added ADAMTS8 in the medium were quantified using ImageJ within the linear range of the measurements, and the amount of the protein remaining in the medium after 8 h incubation was calculated as a percentage of the amount before incubation.

### LRP1-binding assay

Human full-length LRP1 (BioMac, 5 nM in 100 μl of TNC) was coated overnight at 4 °C onto microtiter plates. Wells were blocked with 3% BSA in TNC (1 h; 37 °C) and washed in TNC-B after this and each subsequent step. Wells were then incubated with various concentrations of recombinant ADAMTS5 or ADAMTS8 (0–500 nM) in blocking solution for 3 h at room temperature (RT). Bound proteins were detected using anti-FLAG M2 antibody (5 μg/ml) (1 h; RT) and then with a secondary antibody coupled to HRP (1 h; RT). Hydrolysis of tetramethylbenzidine substrate (KPL) was measured at 450 nm using a FLUOstar Omega (BMG Labtech). Each value was normalized by subtracting the amount of recombinant protein bound to the control well that was not coated with LRP1. Typically, for both enzymes, the noncoated controls gave a signal below 0.1 absorbance unit. This amounted to ≈15% of the maximum ADAMTS5 signal.

### Alpha-2 macroglobulin (α2M) cleavage assay

α2M (250 nM, R&D Systems, Cat. no: 1938-PI-050) was incubated with 250 nM ADAMTS8 or ADAMTS1 at 37 °C for 24 h in TNC-B buffer. Subsequently, reaction products were analyzed by SDS-PAGE under non-reducing conditions (without prior heating) followed by immunoblot analysis using an anti-α2M antibody (R&D Systems, product code α2M).

### Proteoglycan cleavage assays

Aggrecan (667 nM, Sigma-Aldrich Cat. n.: A1960, numbering according to UniProt accession number: P13608) and biglycan (2 μM, Sigma-Aldrich Cat. n.: B8041) from bovine articular cartilage and purified V1 and V1-5GAG (100 nM) were digested with ADAMTS1, 4, 5, and 8 (1–500 nM) in TNC-B buffer at 37 °C for various durations. Aliquots were removed and reactions were stopped at different time points (0–24 h) with ethylenediaminetetraacetic acid (EDTA, 25 mM) in deglycosylation buffer (50 mM sodium acetate, 25 mM Tris HCl pH 8.0) containing 0.1 U/ml chondroitinase ABC (AMS biotechnology Cat. n. AMS.E1028–02) for 16 h at 37 °C. 0.1 U/ml keratanase (endo-beta galactosidase, Cat. n.: G6920, Sigma) was added to aggrecan digests. Samples were electrophoresed under reducing conditions, and cleavage products were detected by immunoblotting with the following antibodies: mouse monoclonal BC-3, detecting aggrecan cleavage at the Glu^392^-Ala^393^ bond (Life Technologies, Cat n.: MA316888, 2 μg/ml); mouse monoclonal 2B6, recognizing the CS stubs left following treatment of proteoglycans with chondroitinase ABC (AMS biotechnology Europe, Cat. no 270432, 1: 100); goat polyclonal anti-biglycan (R&D Systems, Cat. n. AF2667, 0.4 μg/ml); rabbit polyclonal anti-VC, recognizing the versican sequence ^432^VPKDPEAAEARRG^445^, which spans the Glu^441^-Ala^442^ cleavage site (1 μg/ml) (32); rabbit polyclonal anti-DPEEAE neoepitope antibody (Life Technologies, Cat. n. PA1-1748 A, 2 μg/ml), which only detects versikine, the N-terminal versican fragment generated after proteolysis at Glu^441^-Ala^442^.

### Osteopontin cleavage assay

Recombinant human OPN isoform b (R&D Systems, Cat. n.: 1433-OP, 760 nM), which lacks amino acids 58 to 71 compared with isoform a, was incubated with ADAMTS8 (500–50 nM) in TNC-B for 24 h at 37 °C. Proteolysis was stopped by addition of Bolt-TM LDS Sample Buffer, 5% mercaptoethanol, and heating to 95 °C. Digests were subjected to reducing SDS-PAGE and immunoblotting with rabbit polyclonal anti-OPN antibody (Abcam, Cat. n.: Ab8448, 95 μg/ml). Where indicated, 1 μM recombinant human TIMP-1, -2, -3, or -4 (R&D Systems, Cat. n.: 970-TM, 971-TM, 973-TM, 974-TSF) were preincubated with 500 nM ADAMTS8 for 1 h at 37 °C before digestion. Bands were detected with a Chemidoc Touch Imaging system (Bio-Rad), and intensities were measured using Image lab software version 5.2.1. Sequential exposures were analyzed to avoid saturation artifacts. All quantifications were performed on images taken with the same exposure settings and without post-image processing, with the exception of color thresholding applied equally to all images to reduce background signal.

### Identification of OPN and autolytic cleavage sites by high-resolution LC-MS/MS

Autolysis (ADAMTS8) or digestion (OPN) products were analyzed using LC-MS/MS (24). For this, each sample was dried down to less than 5 μl of volume, then diluted with 50 μl of 6 M urea. Proteins were reduced with 10 mM dithiothreitol (DTT) at 55 °C for 15 min, then alkylated with 30 mM iodoacetamide at room temperature in the dark for 30 min. The iodoacetamide was quenched with an additional 30 mM of DTT. Urea was diluted tenfold using 25 mM ammonium bicarbonate. Subsequently, trypsin (trypsin-gold, Promega, Cat. no: V5280) was added at a 1:50 (wt:wt) ratio and incubated at 37 °C overnight. The solution was desalted on a C18 Sep-Pak (Waters) column using 1% trifluoroacetic acid wash buffer. Peptides were eluted in 60:40 ACN: 1% trifluoroacetic acid, vacuum centrifuged until dry, and resuspended in 1% acetic acid for LC-MS/MS. Each digest and corresponding MS analysis was performed in duplicate, resulting in two technical replicates for each condition.

Peptides were analyzed on a Thermo Fisher Scientific Fusion Lumos tribrid mass spectrometer system interfaced with a Thermo Ultimate 3000 nano-UHPLC at the Cleveland Clinic Proteomics and Metabolomics Core Facility. HPLC was performed using a Dionex 15 cm × 75 μm id Acclaim Pepmap C18, 2 μm, 100 Å reversed-phase capillary chromatography column. Trypsin-digested extracts (5 μl) were injected and peptides eluted from the column by an acetonitrile/0.1% formic acid gradient at a flow rate of 0.3 μl/min and introduced in-line into the mass spectrometer over a 90-min gradient. The nanospray ion source was operated at 1.9 kV. The digest was analyzed using a data-dependent method with 35% collision-induced dissociation fragmentation of the most abundant peptides every 3 s and an isolation window of 0.7 m/z for ion-trap MS/MS. Scans were conducted at a maximum resolution of 120,000 for full MS. Dynamic exclusion was enabled with a repeat count of 1, and ions within 10 ppm of the fragmented mass were excluded for 60 s.

The mass spectra were searched against a full human proteome (all reviewed proteins as of November 2019, 42,000 entries) as well as a single OPN database created from the reported sequence (Uniprot accession number: P10451–5, OPN-b) using Proteome Discoverer 2.4 (PD2.4; Thermo Scientific) with semi-tryptic specificity and a maximum of two missed tryptic cleavages. Peptides were identified using a precursor mass tolerance of 10 ppm and fragment mass tolerance of 0.02 Da. Dynamic modifications included oxidation (Met), acetylation (peptide N-terminal), Gln to pyro-Glu cyclization (Gln N-terminal), and phosphorylation (Ser, Thr, Tyr), and the static modification used was carbamidomethylation (Cys). Peptides were validated using a false discovery rate (FDR) of 1% for high confidence peptides and 5% for medium confidence peptides against a decoy database. Chromatographic retention time alignment was used across samples for accurate label-free quantitation comparison and to increase peptide identifications.

Protein and peptide data were analyzed using Microsoft Excel (Microsoft office 2013). Peptides from each protein of interest were sorted as fully tryptic or semi-tryptic based on the presence of a lysine or arginine preceding their N-terminal amino acid residue or as the C-terminal residue in each peptide. Peptide ratios were quantified in PD2.4 using the label-free quantitation method and then were log2 -transformed in Excel to reduce variations between peptides. Log2-transformed ratios were used to calculate a z-score for significance (24). The z-score equals the peptide abundance ratio (WT/EQ) minus the average ratio of all quantified peptides (in both datasets), divided by the standard deviation of all quantified peptide ratios (in both datasets). The z-score represents the number of standard deviations each peptide ratio is from the mean. Z-scores were calculated for semi-tryptic peptides and tryptic peptides separately. Z-scores and peptide ratios (non-transformed) were visualized as scatter plots to identify semi-tryptic peptides that were more abundant in the WT ADAMTS8 digest compared with the control digest with the active site mutant (E364Q) and tryptic peptides that were more abundant in the control digest. A z-score >2 was considered significant.

### Statistical analysis

Data are presented as mean ± SEM from at least three independent experiments and were analyzed by GraphPad Prism Software. For endocytosis assays, data are represented as mean ± SD. Statistical analysis was performed using the Mann–Whitney test and *p* < 0.05 was considered significant.

## Data availability

Reagents and data presented in this study are available from the corresponding author upon request.

The mass spectrometry proteomics data have been deposited to the ProteomeXchange Consortium *via* the PRIDE (64) partner repository with the dataset identifier PXD027724 and 10.6019/PXD027724.

## Supporting information

This article contains supporting information (65, 66, 67, 68, 69, 70, 71, 72, 73, 74, 75).

## Conflict of interest

The authors declare that they have no conflicts of interest with the contents of this article.
