# Supplementary material for: Post-translational regulation and proteolytic activity of the metalloproteinase ADAMTS8
Source: J Biol Chem. 2021 Oct 21;297(5):101323. doi: 10.1016/j.jbc.2021.101323 (PMC8577114; doi:10.1016/j.jbc.2021.101323)
Supplement: Figures S1–S5 and Tables S1–S3 [file mmc1.pdf]

SUPPORTING INFORMATION

POST-TRANSLATIONAL REGULATION AND PROTEOLYTIC ACTIVITY OF THE METALLOPROTEINASE ADAMTS8

Salvatore Santamaria[1,*], Daniel R. Martin,[2] Xiangyi Dong[1], Kazuhiro Yamamoto,[3] Suneel S. Apte,[2] and Josefin Ahnström[1]

From the Department of Immunology and Inflammation, Imperial College London, Du Cane Road W12 0NN, London, UK; [2]Department of Biomedical Engineering, Cleveland Clinic Lerner Research Institute, Cleveland OH 44195, USA; [3]Institute of Life Course and Medical Sciences, University of Liverpool, William Duncan Building, 6 West Derby Street, L7 8TX, Liverpool, UK.

* to whom the correspondence should be addressed: Salvatore Santamaria, Department of Immunology and Inflammation, 5th Floor Commonwealth Building, Hammersmith Hospital Campus, Du Cane Road, W12 0NN, London, United Kingdom; s.santamaria@imperial.ac.uk; Tel: ++44 (0) 20 83 83 22 98

Running title: ADAMTS-8 expression and proteolytic activity

List:
Supporting Figures: S1-S5
Supporting Tables: S1-S3

Fig. S1. Annotated MS/MS spectrum of ADAMTS8 autolysis peptides. Parent ions were selected for collision-induced dissociation fragmentation at 35% collisional energy. This fragmentation creates breakages at the amino-carboxyl bond to generate a series of b-ions (which retain the original N-terminus (in blue)) and y-ions (which retain the original C-terminus (in red)). The m/z of these ions corresponds to known amino acid residues and allow confirmation of the parent ion amino acid sequence (labelled on each spectrum).

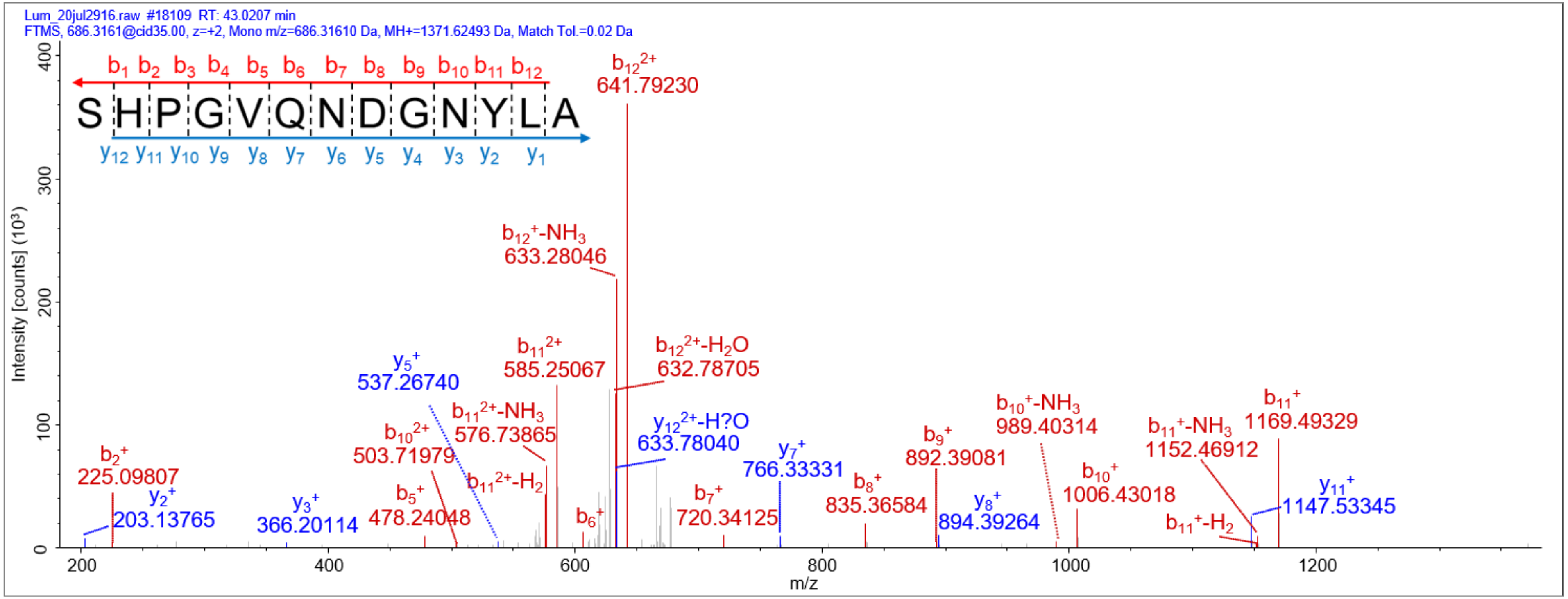

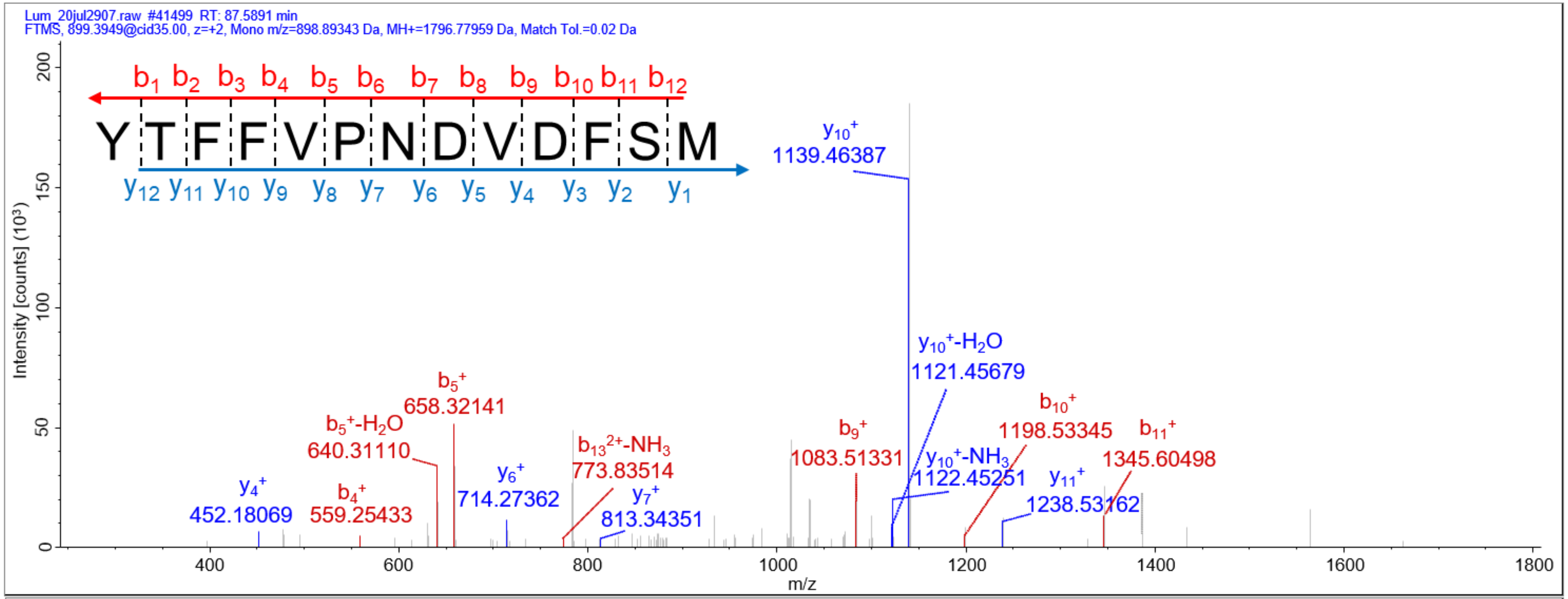
Lum_20jul2907.raw #41499 RT: 87.5891 min
FTMS, 899.3949@cid35.00, z=+2, Mono m/z=898.89343 Da, MH+=1796.77959 Da, Match Tol.=0.02 Da
b1 b2 b3 b4 b5 b6 b7 b8 b9 b10 b11 b12
Y T F F V P N D V D F S M
y12 y11 y10 y9 y8 y7 y6 y5 y4 y3 y2 y1
y10+ 1139.46387
y10+-H2O 1121.45679
b5+ 658.32141
b5+-H2O 640.31110
b13 2+-NH3 773.83514
b10+ 1198.53345
b11+ 1345.60498
b9+ 1083.51331
y10+-NH3 1122.45251
y4+ 452.18069
b4+ 559.25433
y6+ 714.27362
y7+ 813.34351
y11+ 1238.53162
Intensity [counts] (10³)
m/z

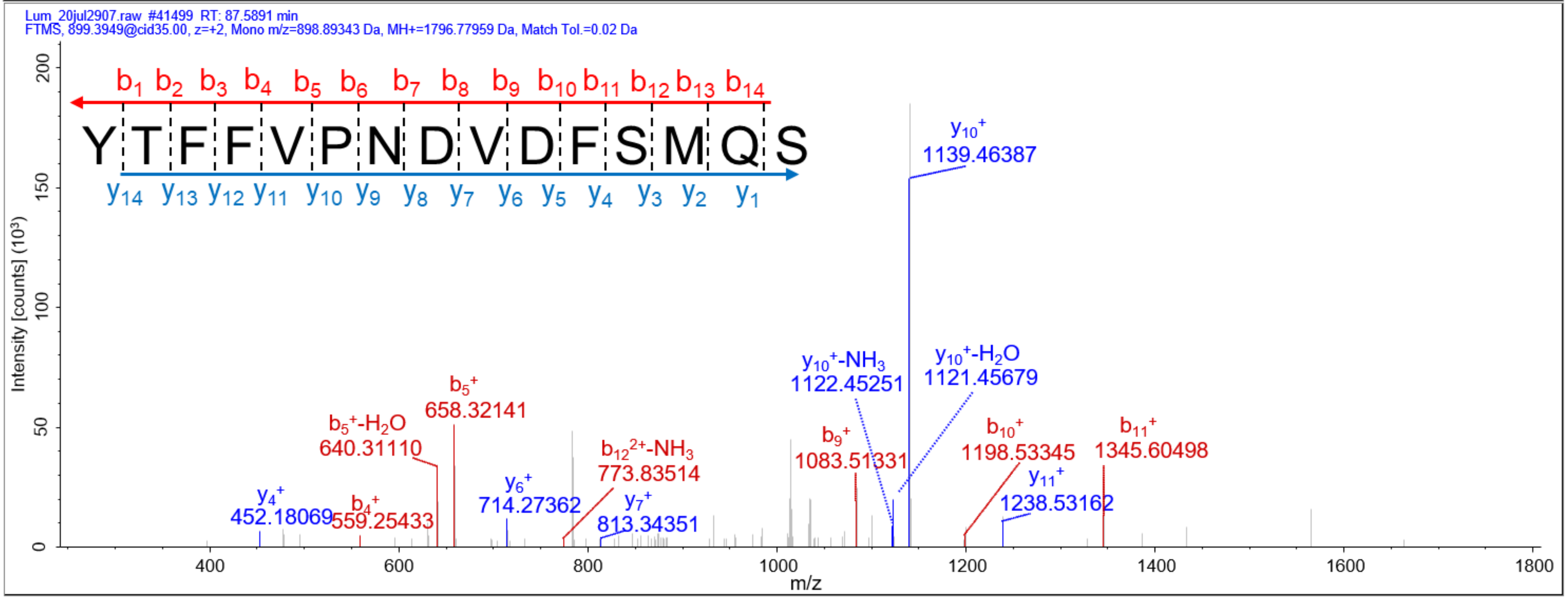
Lum_20jul2907.raw #41499 RT: 87.5891 min
FTMS, 899.3949@cid35.00, z=+2, Mono m/z=898.89343 Da, MH+=1796.77959 Da, Match Tol.=0.02 Da
b1 b2 b3 b4 b5 b6 b7 b8 b9 b10 b11 b12 b13 b14
Y T F F V P N D V D F S M Q S
y14 y13 y12 y11 y10 y9 y8 y7 y6 y5 y4 y3 y2 y1
y10+ 1139.46387
y10+-NH3 1122.45251
y10+-H2O 1121.45679
b5+ 658.32141
b5+-H2O 640.31110
b12 2+-NH3 773.83514
b9+ 1083.51331
b10+ 1198.53345
b11+ 1345.60498
y4+ 452.18069
b4+ 559.25433
y6+ 714.27362
y7+ 813.34351
y11+ 1238.53162
Intensity [counts] (10³)
m/z

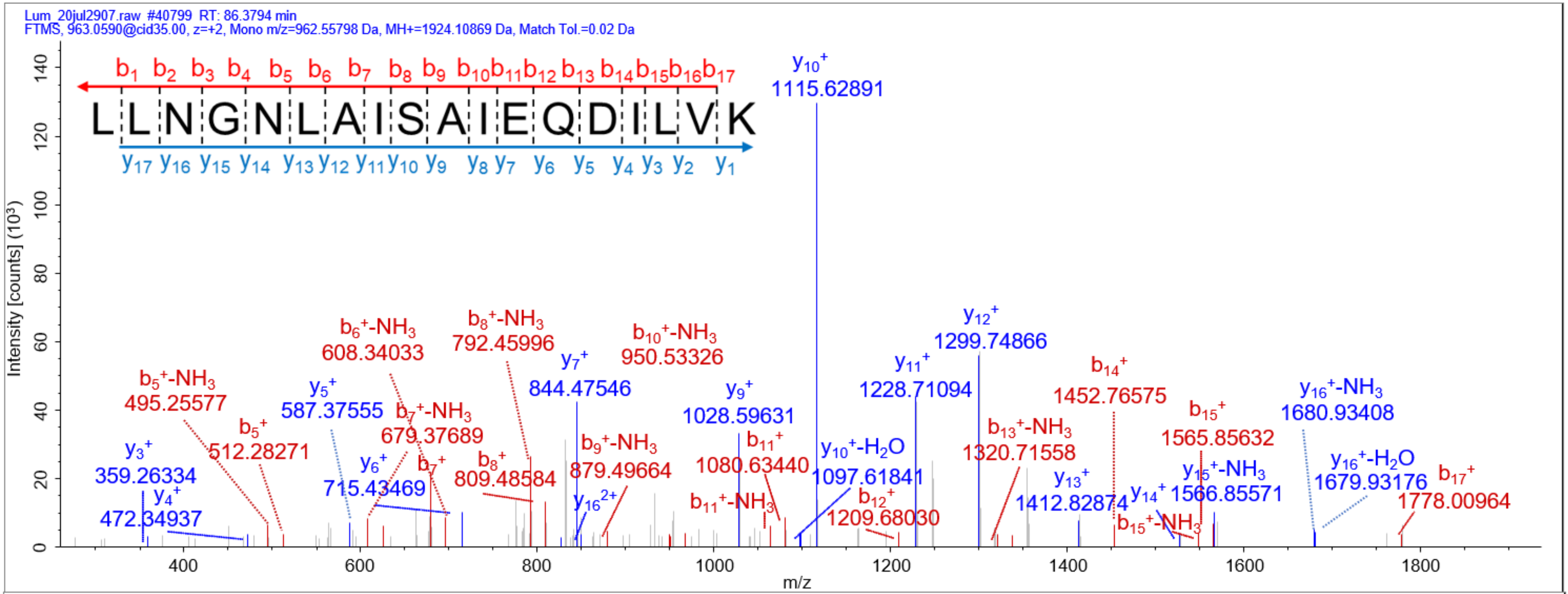

Lum_20jul2907.raw #40799 RT: 86.3794 min
FTMS, 963.0590@cid35.00, z=+2, Mono m/z=962.55798 Da, MH+=1924.10869 Da, Match Tol.=0.02 Da
L L N G N L A I S A I E Q D I L V K
y10+ 1115.62891
y12+ 1299.74866
y11+ 1228.71094
y9+ 1028.59631
y7+ 844.47546
y5+ 587.37555
y6+ 715.43469
y3+ 359.26334
y4+ 472.34937
b5+-NH3 495.25577
b5+ 512.28271
b6+-NH3 608.34033
b7+-NH3 679.37689
b7+
b8+-NH3 792.45996
b8+ 809.48584
b9+-NH3 879.49664
b10+-NH3 950.53326
y16 2+
b11+ 1080.63440
b11+-NH3
y10+-H2O 1097.61841
b12+ 1209.68030
b13+-NH3 1320.71558
y13+ 1412.82874
b14+ 1452.76575
b15+ 1565.85632
y14+
b15+-NH3
y15+-NH3 1566.85571
y16+-NH3 1680.93408
y16+-H2O 1679.93176
b17+ 1778.00964
Intensity [counts] (10^3)
m/z

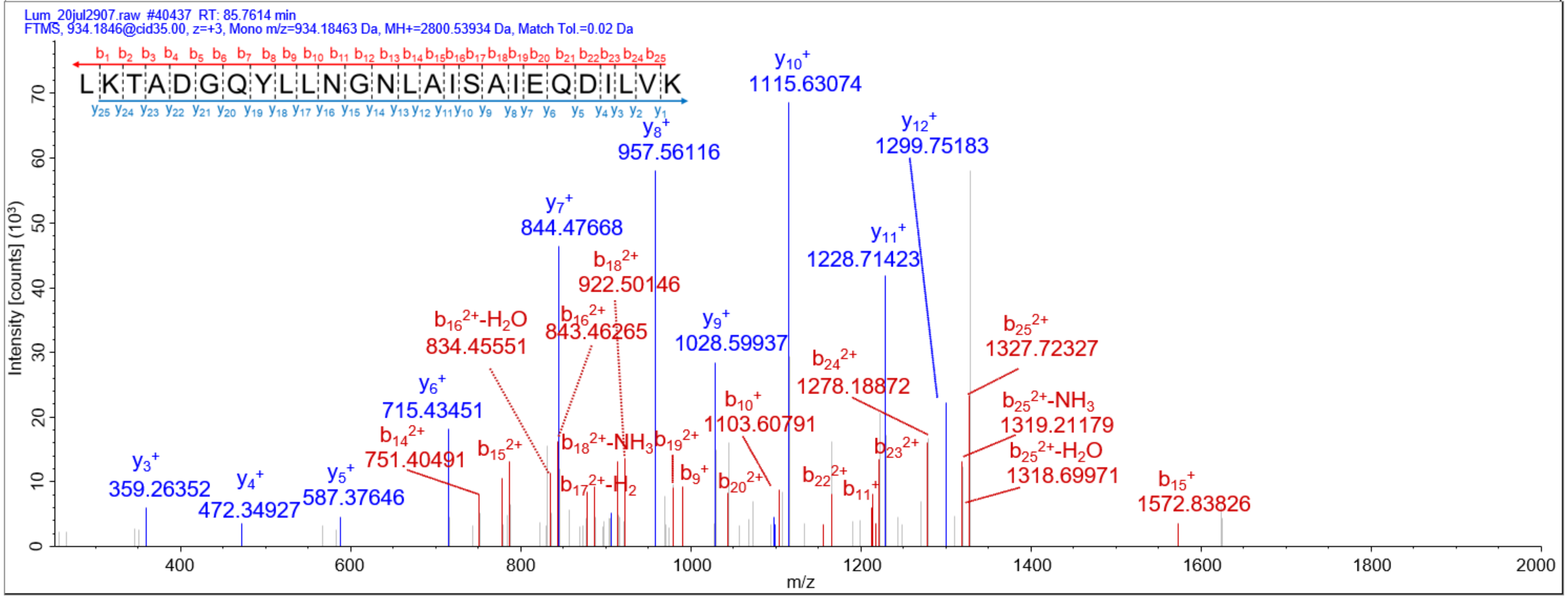

Lum_20jul2907.raw #40437 RT: 85.7614 min
FTMS, 934.1846@cid35.00, z=+3, Mono m/z=934.18463 Da, MH+=2800.53934 Da, Match Tol.=0.02 Da
L K T A D G Q Y L L N G N L A I S A I E Q D I L V K
y10+ 1115.63074
y8+ 957.56116
y12+ 1299.75183
y7+ 844.47668
y11+ 1228.71423
b18 2+ 922.50146
b16 2+-H2O 834.45551
b16 2+ 843.46265
y9+ 1028.59937
b25 2+ 1327.72327
b24 2+ 1278.18872
y6+ 715.43451
b10+ 1103.60791
b25 2+-NH3 1319.21179
b14 2+ 751.40491
b15 2+
b18 2+-NH3
b19 2+
b23 2+
b25 2+-H2O 1318.69971
y3+ 359.26352
y4+ 472.34927
y5+ 587.37646
b17 2+-H2
b9+
b20 2+
b22 2+
b11+
b15+ 1572.83826
Intensity [counts] (10^3)
m/z

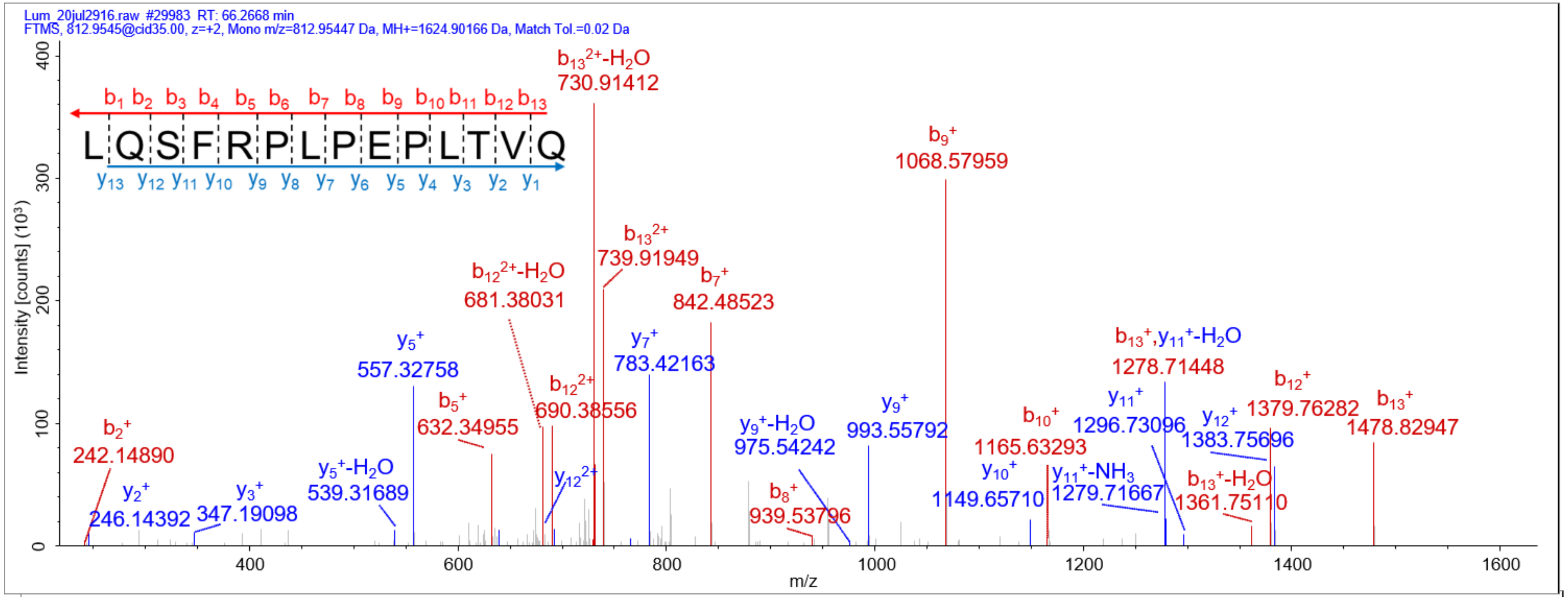

Lum_20jul2916.raw #29983 RT: 66.2668 min
FTMS, 812.9545@cid35.00, z=+2, Mono m/z=812.95447 Da, MH+=1624.90166 Da, Match Tol.=0.02 Da
L Q S F R P L P E P L T V Q
b1 b2 b3 b4 b5 b6 b7 b8 b9 b10 b11 b12 b13
y13 y12 y11 y10 y9 y8 y7 y6 y5 y4 y3 y2 y1
b13 2+-H2O 730.91412
b9+ 1068.57959
b13 2+ 739.91949
b12 2+-H2O 681.38031
b7+ 842.48523
y5+ 557.32758
y7+ 783.42163
b13+,y11+-H2O 1278.71448
b12 2+ 690.38556
b5+ 632.34955
b12+ 1379.76282
b13+ 1478.82947
y11+ 1296.73096
y12+ 1383.75696
b2+ 242.14890
y9+-H2O 975.54242
y9+ 993.55792
b10+ 1165.63293
y5+-H2O 539.31689
y12 2+
y10+ 1149.65710
y11+-NH3 1279.71667
b13+-H2O 1361.75110
y2+ 246.14392
y3+ 347.19098
b8+ 939.53796
Intensity [counts] (10³)
m/z

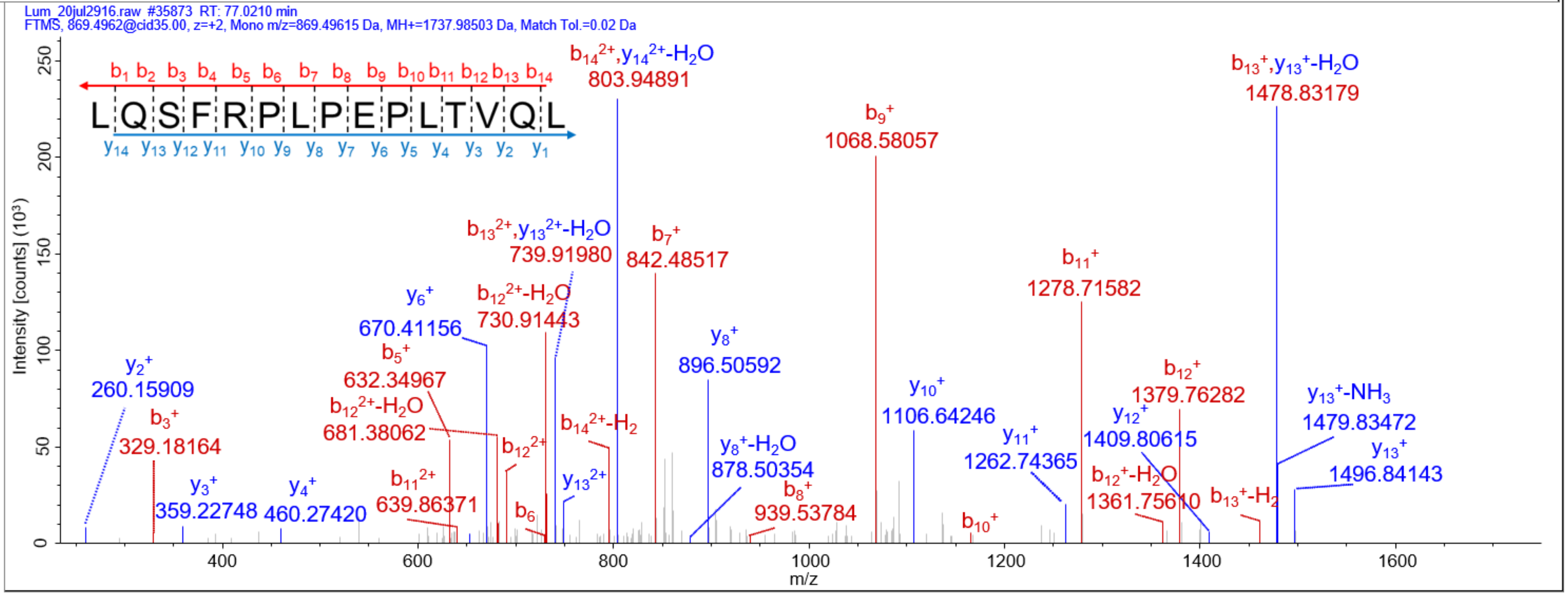

Lum_20jul2916.raw #35873 RT: 77.0210 min
FTMS, 869.4962@cid35.00, z=+2, Mono m/z=869.49615 Da, MH+=1737.98503 Da, Match Tol.=0.02 Da
L Q S F R P L P E P L T V Q L
b1 b2 b3 b4 b5 b6 b7 b8 b9 b10 b11 b12 b13 b14
y14 y13 y12 y11 y10 y9 y8 y7 y6 y5 y4 y3 y2 y1
b14 2+,y14 2+-H2O 803.94891
b13+,y13+-H2O 1478.83179
b9+ 1068.58057
b13 2+,y13 2+-H2O 739.91980
b7+ 842.48517
b11+ 1278.71582
y6+ 670.41156
b12 2+-H2O 730.91443
b5+ 632.34967
y8+ 896.50592
y2+ 260.15909
y10+ 1106.64246
b12+ 1379.76282
y13+-NH3 1479.83472
b3+ 329.18164
b12 2+-H2O 681.38062
b14 2+-H2
b12 2+
y12+ 1409.80615
y8+-H2O 878.50354
y11+ 1262.74365
y13+ 1496.84143
y13 2+
b11 2+ 639.86371
b12+-H2O 1361.75610
y3+ 359.22748
y4+ 460.27420
b8+ 939.53784
b13+-H2
b6
b10+
Intensity [counts] (10³)
m/z

Fig. S2. ADAMTS8 does not bind to LRP1. Human full-length LRP1 (5 nM) was coated overnight. Wells were blocked with 3% BSA in TNC and washed in TNC-B after this and each subsequent step. Wells were then incubated with various concentrations of recombinant ADAMTS5 or ADAMTS8 (0-500 nM) in blocking solution for 3 h at room temperature (RT). Bound proteins were detected using anti-FLAG M2 antibody and then with a secondary antibody coupled to HRP. Hydrolysis of tetramethylbenzidine substrate was measured at 450 nm. Each value was normalized by subtracting the amount of recombinant protein bound to control well that was not coated with LRP1.

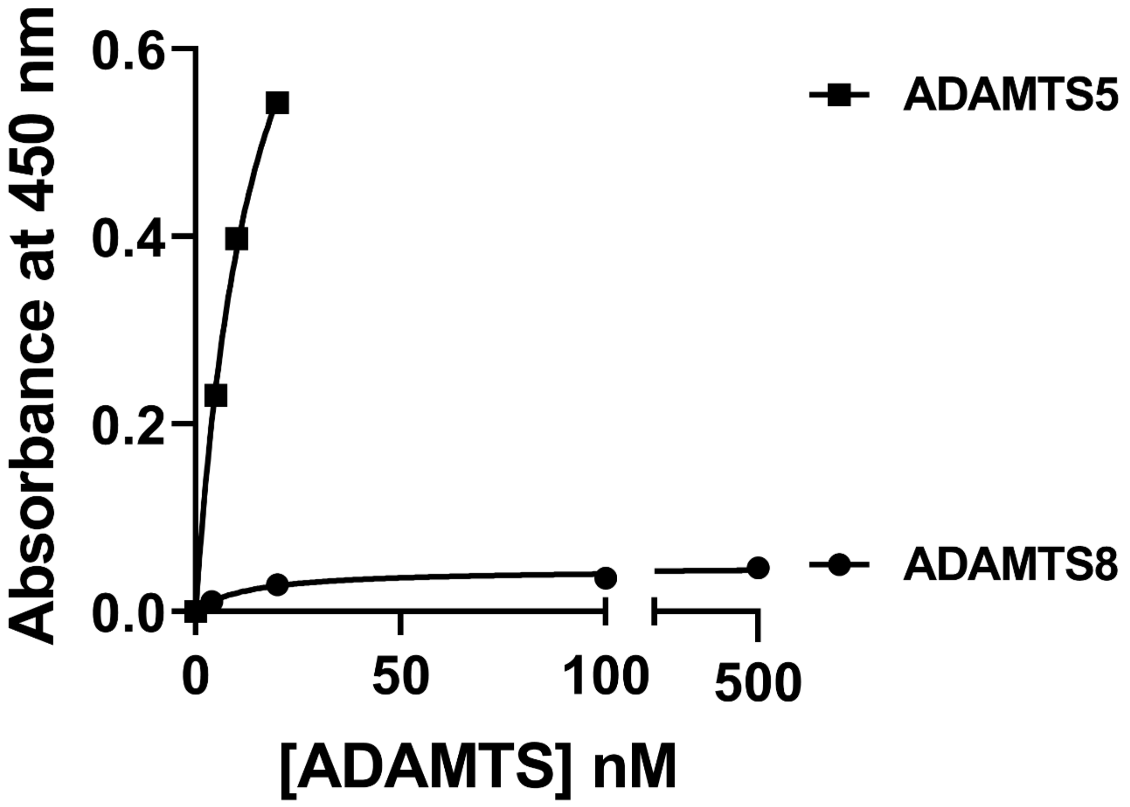

Fig. S3. Annotated MS/MS spectra of ADAMTS8 derived peptides from the 2-hour OPN digestion. Parent ions were selected for collision-induced dissociation fragmentation at 35% collisional energy. This fragmentation creates breakages at the amino-carboxyl bond to generate a series of b-ions (which retain the original N-terminus, in blue) and y-ions (which retain the original C-terminus, in red)). The m/z of the b/y ions corresponds to known amino acid residues or amino acid ensembles, providing the parent ion amino acid sequence (labelled on each spectrum).

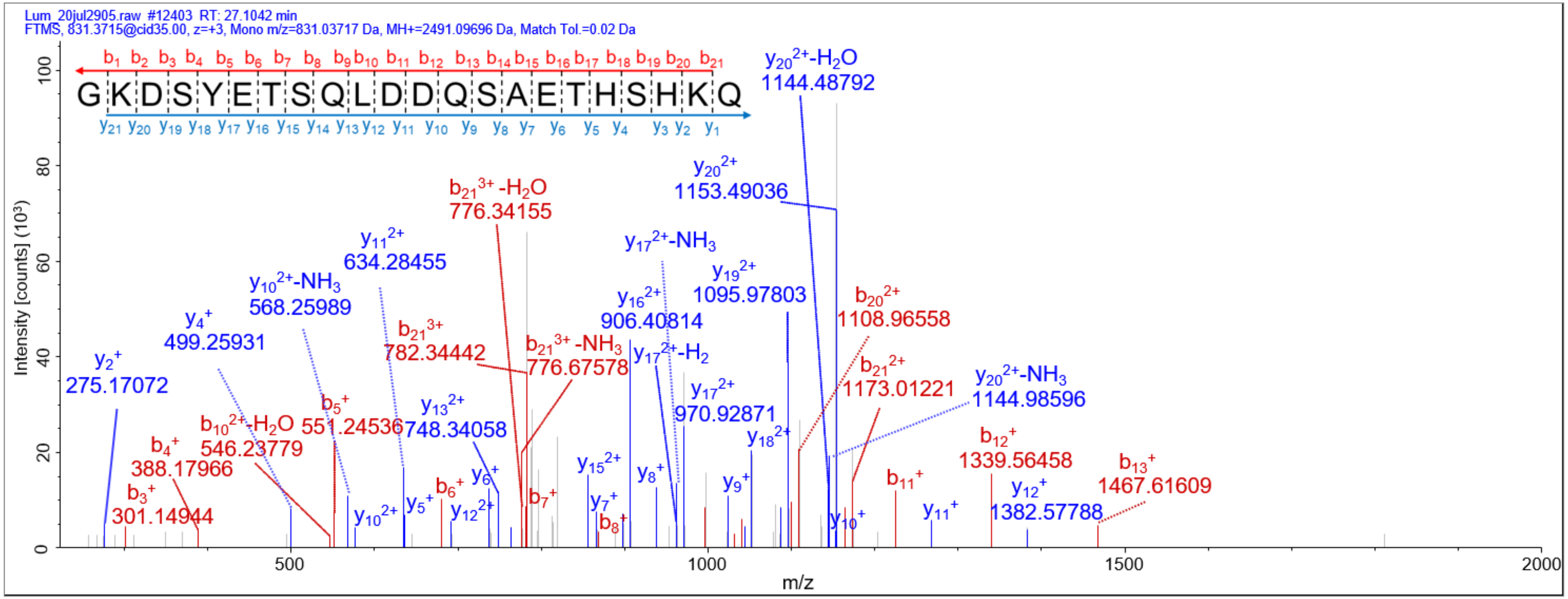

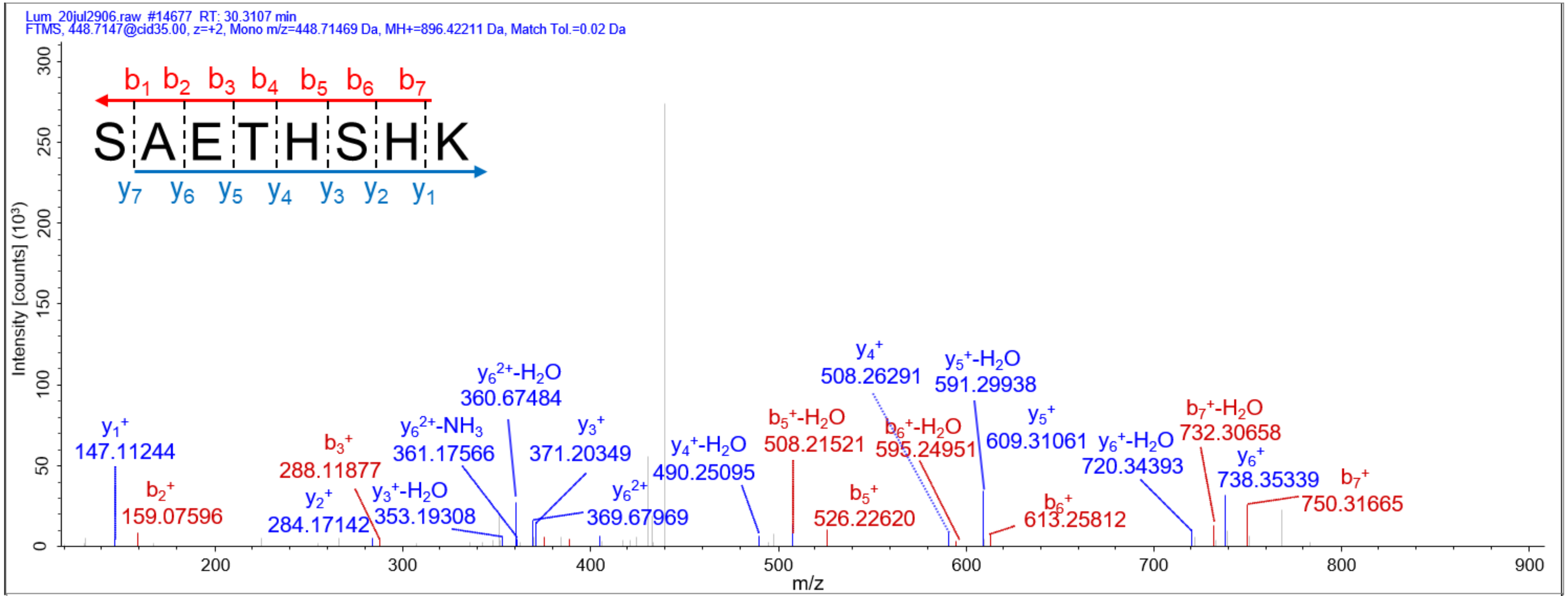
Lum_20jul2906.raw #14677 RT: 30.3107 min
FTMS, 448.7147@cid35.00, z=+2, Mono m/z=448.71469 Da, MH+=896.42211 Da, Match Tol.=0.02 Da
b1 b2 b3 b4 b5 b6 b7
S A E T H S H K
y7 y6 y5 y4 y3 y2 y1
Intensity [counts] (10^3)
m/z
y1+ 147.11244
b2+ 159.07596
y2+ 284.17142
b3+ 288.11877
y3+-H2O 353.19308
y6 2+-NH3 361.17566
y6 2+-H2O 360.67484
y3+ 371.20349
y6 2+ 369.67969
y4+-H2O 490.25095
y4+ 508.26291
b5+-H2O 508.21521
b5+ 526.22620
y5+-H2O 591.29938
b6+-H2O 595.24951
y5+ 609.31061
b6+ 613.25812
y6+-H2O 720.34393
b7+-H2O 732.30658
y6+ 738.35339
b7+ 750.31665

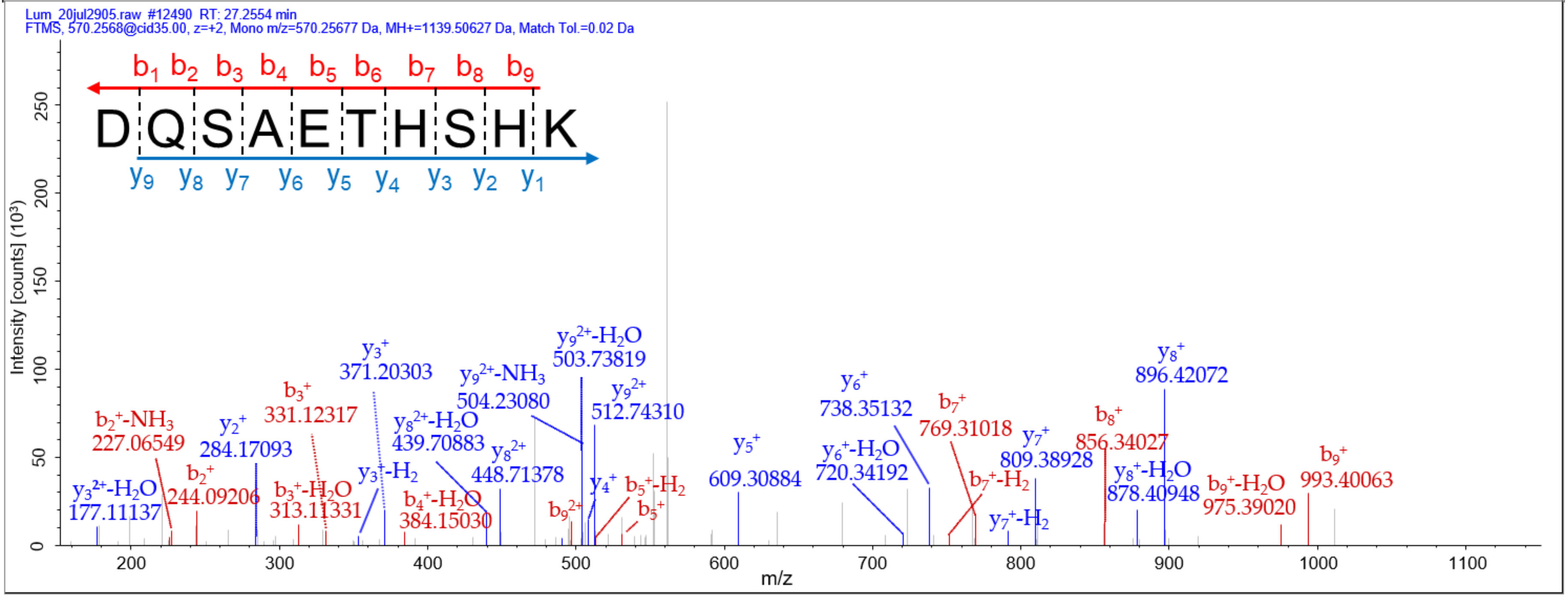
Lum_20jul2905.raw #12490 RT: 27.2554 min
FTMS, 570.2568@cid35.00, z=+2, Mono m/z=570.25677 Da, MH+=1139.50627 Da, Match Tol.=0.02 Da
b1 b2 b3 b4 b5 b6 b7 b8 b9
D Q S A E T H S H K
y9 y8 y7 y6 y5 y4 y3 y2 y1
Intensity [counts] (10^3)
m/z
y3 2+-H2O 177.11137
b2+-NH3 227.06549
b2+ 244.09206
y2+ 284.17093
b3+ 331.12317
b3+-H2O 313.11331
y3+ 371.20303
y3+-H2
b4+-H2O 384.15030
y8 2+-H2O 439.70883
y8 2+ 448.71378
y9 2+-NH3 504.23080
y9 2+-H2O 503.73819
y9 2+ 512.74310
b9 2+
y4+
b5+-H2
b5+
y5+ 609.30884
y6+ 738.35132
y6+-H2O 720.34192
b7+ 769.31018
b7+-H2
y7+ 809.38928
y7+-H2
b8+ 856.34027
y8+-H2O 878.40948
y8+ 896.42072
b9+-H2O 975.39020
b9+ 993.40063

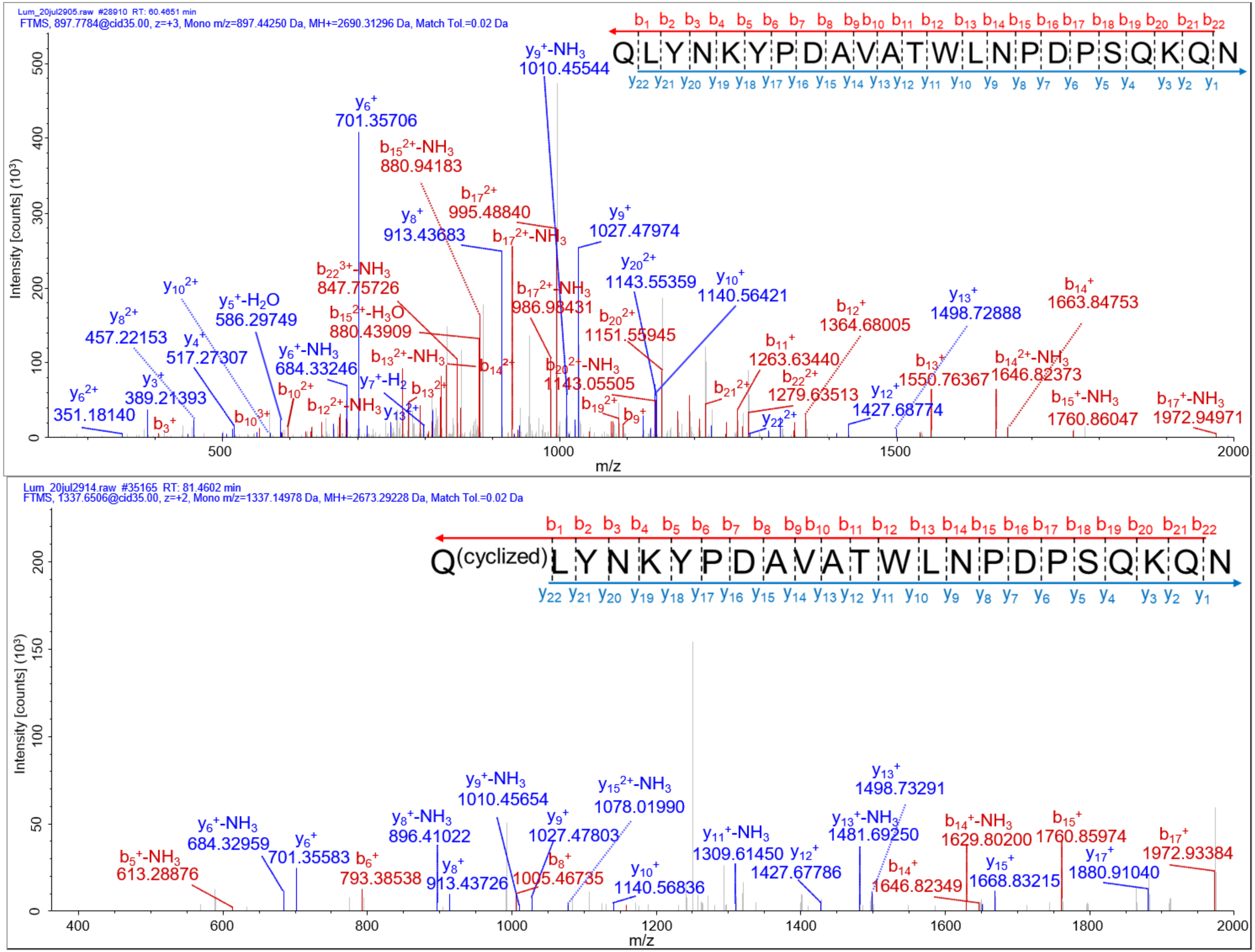

Lum_20jul2905.raw #28910 RT: 60.4651 min
FTMS, 897.7784@cid35.00, z=+3, Mono m/z=897.44250 Da, MH+=2690.31296 Da, Match Tol.=0.02 Da
b1 b2 b3 b4 b5 b6 b7 b8 b9 b10 b11 b12 b13 b14 b15 b16 b17 b18 b19 b20 b21 b22
Q L Y N K Y P D A V A T W L N P D P S Q K Q N
y22 y21 y20 y19 y18 y17 y16 y15 y14 y13 y12 y11 y10 y9 y8 y7 y6 y5 y4 y3 y2 y1
Intensity [counts] (10³)
m/z
y9+-NH3 1010.45544
y6+ 701.35706
b15 2+-NH3 880.94183
b17 2+ 995.48840
y8+ 913.43683
b17 2+-NH3
y9+ 1027.47974
b22 3+-NH3 847.75726
b17 2+-NH3 986.98431
y20 2+ 1143.55359
y10+ 1140.56421
y10 2+
y5+-H2O 586.29749
b15 2+-H3O 880.43909
b20 2+ 1151.55945
b12+ 1364.68005
y13+ 1498.72888
b14+ 1663.84753
y8 2+ 457.22153
y4+ 517.27307
y6+-NH3 684.33246
b13 2+-NH3
b14 2+
b20 2+-NH3 1143.05505
b11+ 1263.63440
b13+ 1550.76367
b14 2+-NH3 1646.82373
y3+ 389.21393
y6 2+ 351.18140
b3+
b10 3+
b10 2+
b12 2+-NH3
y7+-H2
y13 2+
b13 2+
b19 2+
b9+
b21 2+
b22 2+ 1279.63513
y12+ 1427.68774
y22 2+
b15+-NH3 1760.86047
b17+-NH3 1972.94971
Lum_20jul2914.raw #35165 RT: 81.4602 min
FTMS, 1337.6506@cid35.00, z=+2, Mono m/z=1337.14978 Da, MH+=2673.29228 Da, Match Tol.=0.02 Da
Q(cyclized) L Y N K Y P D A V A T W L N P D P S Q K Q N
y9+-NH3 1010.45654
y15 2+-NH3 1078.01990
y13+ 1498.73291
y6+-NH3 684.32959
y8+-NH3 896.41022
y9+ 1027.47803
y11+-NH3 1309.61450
y13+-NH3 1481.69250
b14+-NH3 1629.80200
b15+ 1760.85974
b17+ 1972.93384
b5+-NH3 613.28876
y6+ 701.35583
b6+ 793.38538
y8+ 913.43726
b8+ 1005.46735
y10+ 1140.56836
y12+ 1427.67786
b14+ 1646.82349
y15+ 1668.83215
y17+ 1880.91040

Fig. S4. Tryptic peptides detected in A) 2 h and B) 24 digestions of OPN with WT and EQ ADAMTS8. A z-score > 2 was considered significant.

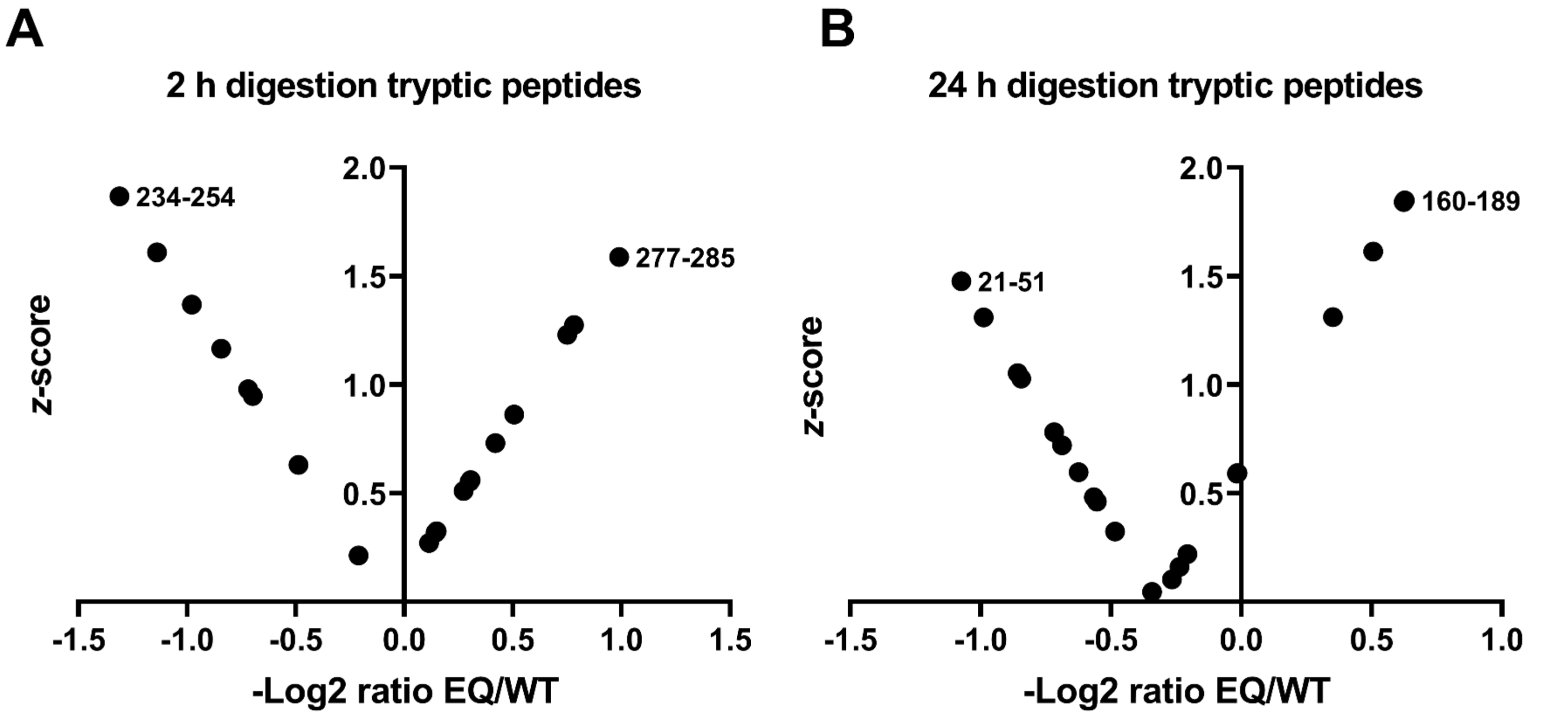

Fig. S5. Annotated MS/MS spectra of ADAMTS8 derived peptides from the 24-hour OPN digestion. Parent ions were selected for collision-induced dissociation fragmentation at 35% collisional energy. This fragmentation creates breakages at the amino-carboxyl bond to generate a series of b-ions (which retain the original N-terminus, in blue) and y-ions (which retain the original C-terminus, in red). The m/z of the b/y ions corresponds to known amino acid residues or amino acid ensembles, providing the parent ion amino acid sequence (labelled on each spectrum).

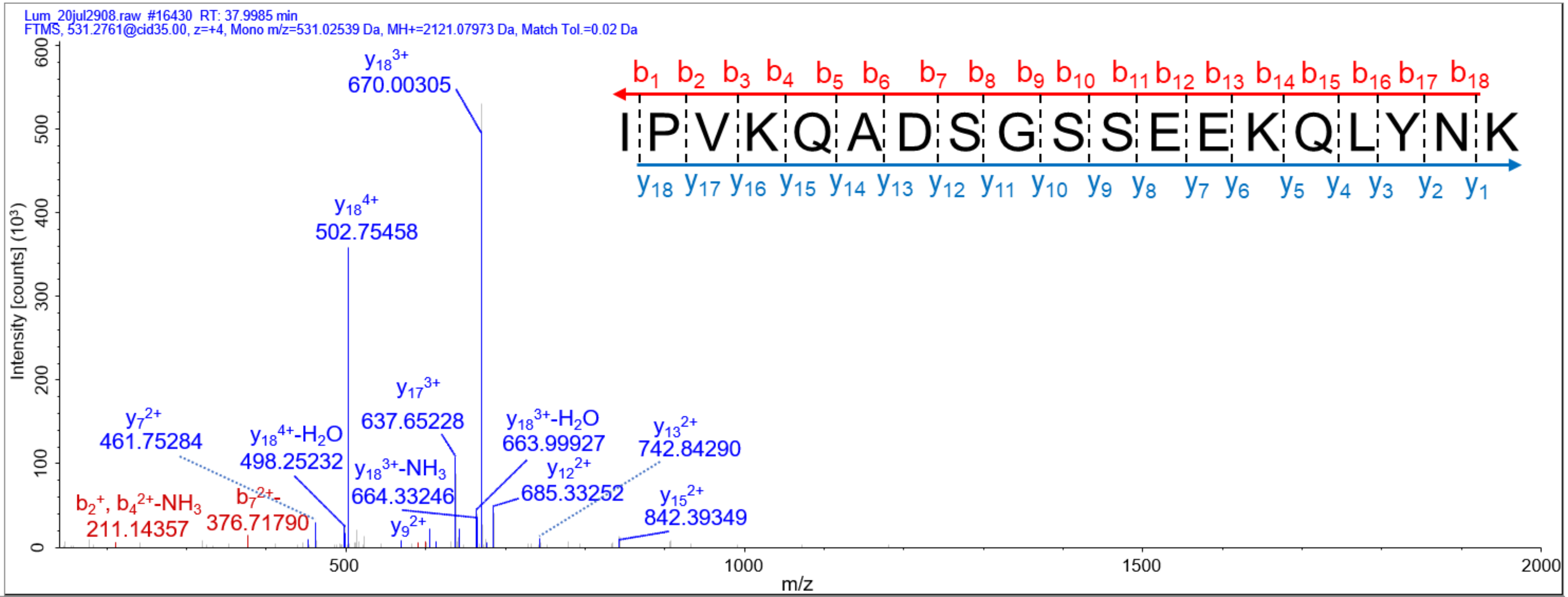

Lum_20jul2908.raw #16430 RT: 37.9985 min
FTMS, 531.2761@cid35.00, z=+4, Mono m/z=531.02539 Da, MH+=2121.07973 Da, Match Tol.=0.02 Da
Intensity [counts] (10³)
m/z
b1 b2 b3 b4 b5 b6 b7 b8 b9 b10 b11 b12 b13 b14 b15 b16 b17 b18
I P V K Q A D S G S S E E K Q L Y N K
y18 y17 y16 y15 y14 y13 y12 y11 y10 y9 y8 y7 y6 y5 y4 y3 y2 y1
y18 3+ 670.00305
y18 4+ 502.75458
y17 3+ 637.65228
y7 2+ 461.75284
y18 4+-H2O 498.25232
y18 3+-H2O 663.99927
y13 2+ 742.84290
y18 3+-NH3 664.33246
y12 2+ 685.33252
y15 2+ 842.39349
b2 +, b4 2+-NH3 211.14357
b7 2+ 376.71790
y9 2+

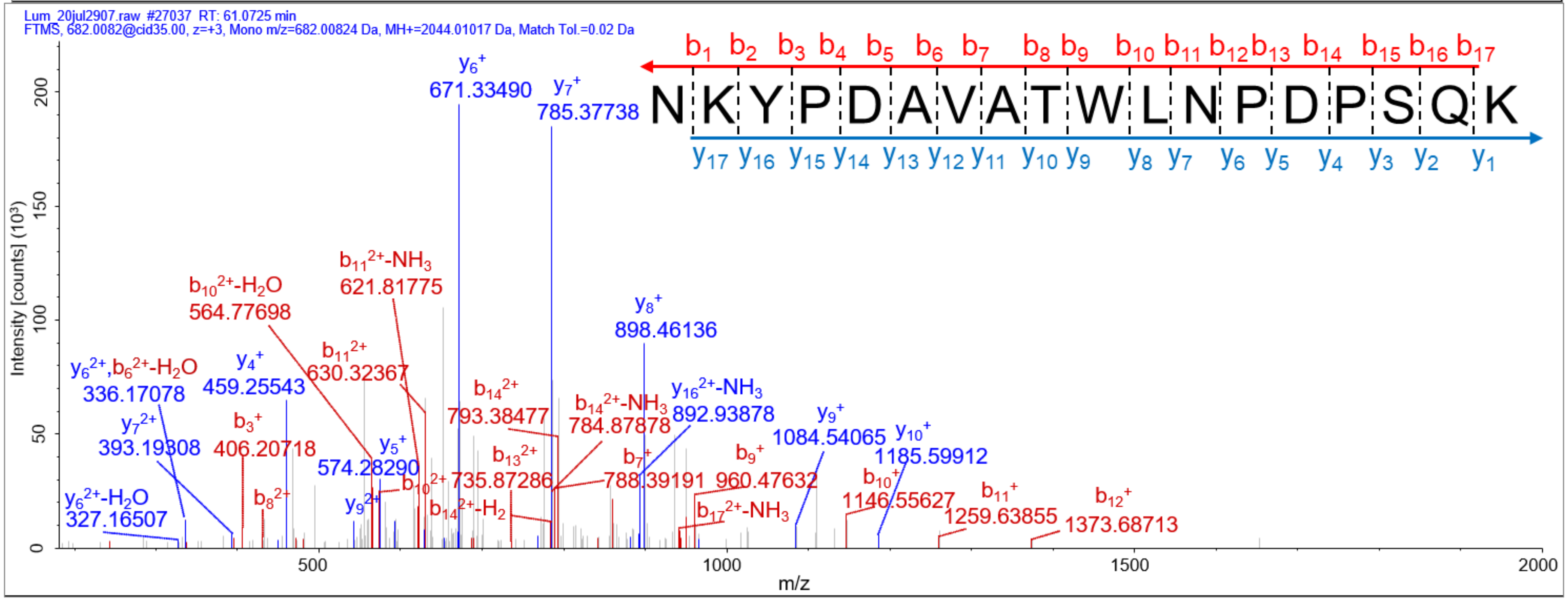

Lum_20jul2907.raw #27037 RT: 61.0725 min
FTMS, 682.0082@cid35.00, z=+3, Mono m/z=682.00824 Da, MH+=2044.01017 Da, Match Tol.=0.02 Da
Intensity [counts] (10³)
m/z
b1 b2 b3 b4 b5 b6 b7 b8 b9 b10 b11 b12 b13 b14 b15 b16 b17
N K Y P D A V A T W L N P D P S Q K
y17 y16 y15 y14 y13 y12 y11 y10 y9 y8 y7 y6 y5 y4 y3 y2 y1
y6 + 671.33490
y7 + 785.37738
b11 2+-NH3 621.81775
b10 2+-H2O 564.77698
y8 + 898.46136
b11 2+ 630.32367
y6 2+,b6 2+-H2O 336.17078
y4 + 459.25543
b14 2+ 793.38477
b14 2+-NH3 784.87878
y16 2+-NH3 892.93878
y9 + 1084.54065
y10 + 1185.59912
y7 2+ 393.19308
b3 + 406.20718
y5 + 574.28290
b13 2+ 735.87286
b7 + 788.39191
b9 + 960.47632
b10 + 1146.55627
b11 + 1259.63855
b12 + 1373.68713
y6 2+-H2O 327.16507
b8 2+
y9 2+
b10 2+
b14 2+-H2
b17 2+-NH3

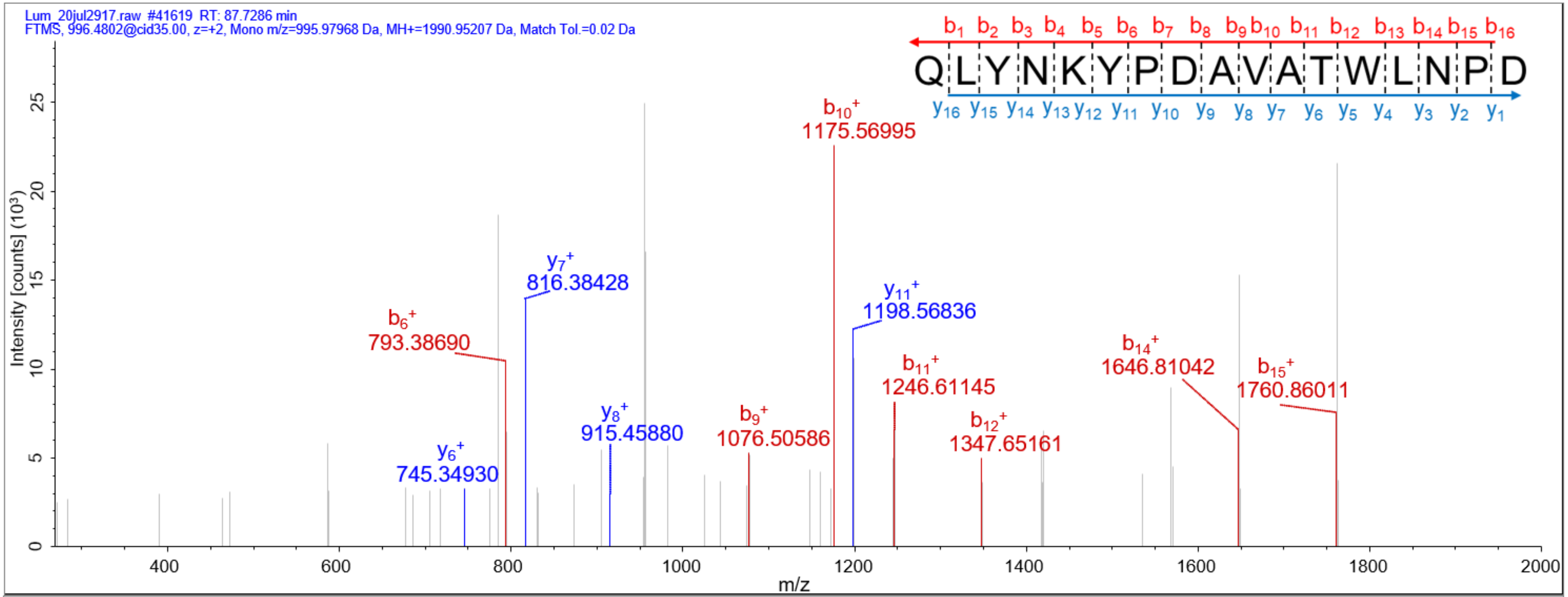
Lum_20jul2917.raw #41619 RT: 87.7286 min
FTMS, 996.4802@cid35.00, z=+2, Mono m/z=995.97968 Da, MH+=1990.95207 Da, Match Tol.=0.02 Da
Q L Y N K Y P D A V A T W L N P D
b1 b2 b3 b4 b5 b6 b7 b8 b9 b10 b11 b12 b13 b14 b15 b16
y16 y15 y14 y13 y12 y11 y10 y9 y8 y7 y6 y5 y4 y3 y2 y1
Intensity [counts] (10³)
b10+ 1175.56995
y7+ 816.38428
y11+ 1198.56836
b6+ 793.38690
b11+ 1246.61145
b14+ 1646.81042
b15+ 1760.86011
y8+ 915.45880
b9+ 1076.50586
b12+ 1347.65161
y6+ 745.34930
m/z

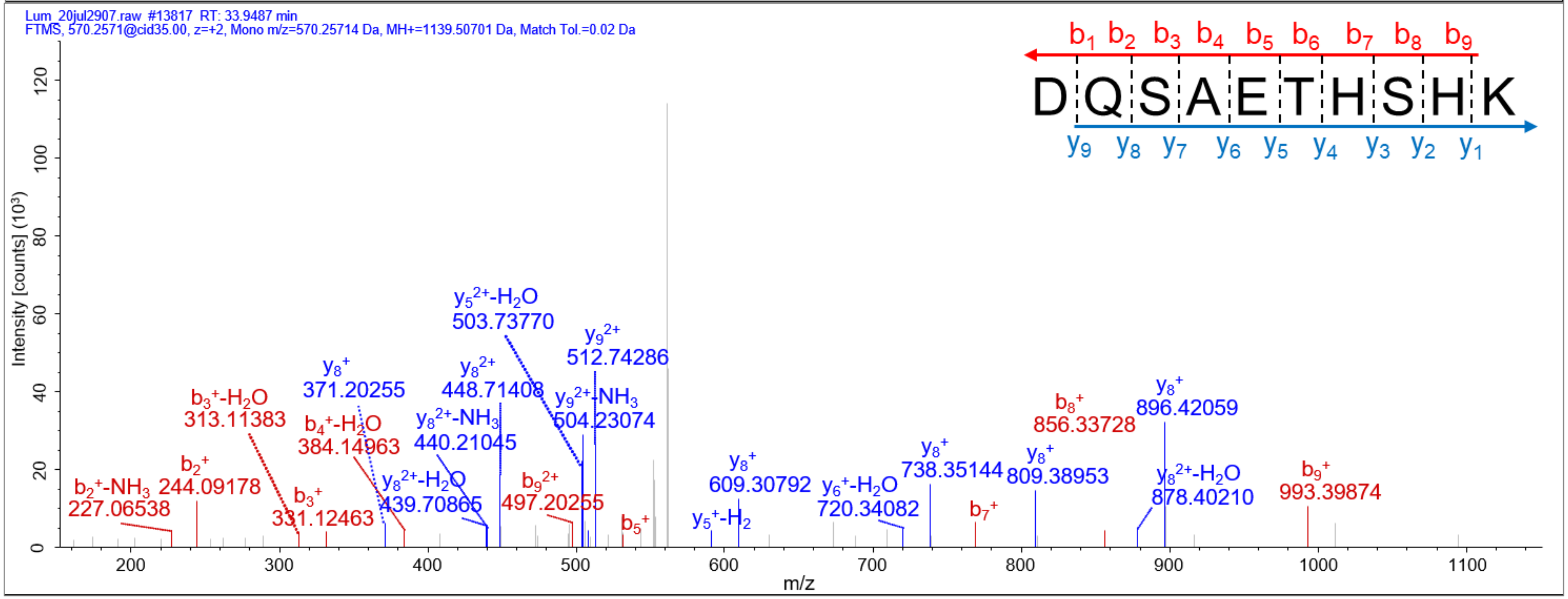
Lum_20jul2907.raw #13817 RT: 33.9487 min
FTMS, 570.2571@cid35.00, z=+2, Mono m/z=570.25714 Da, MH+=1139.50701 Da, Match Tol.=0.02 Da
D Q S A E T H S H K
b1 b2 b3 b4 b5 b6 b7 b8 b9
y9 y8 y7 y6 y5 y4 y3 y2 y1
Intensity [counts] (10³)
y5 2+-H2O 503.73770
y9 2+ 512.74286
y8+ 371.20255
y8 2+ 448.71408
y9 2+-NH3 504.23074
b3+-H2O 313.11383
b4+-H2O 384.14963
y8 2+-NH3 440.21045
b8+ 856.33728
y8+ 896.42059
b2+ 244.09178
b2+-NH3 227.06538
b3+ 331.12463
y8 2+-H2O 439.70865
b9 2+ 497.20255
b5+
y5+-H2
y8+ 609.30792
y6+-H2O 720.34082
y8+ 738.35144
y8+ 809.38953
b7+
y8 2+-H2O 878.40210
b9+ 993.39874
m/z

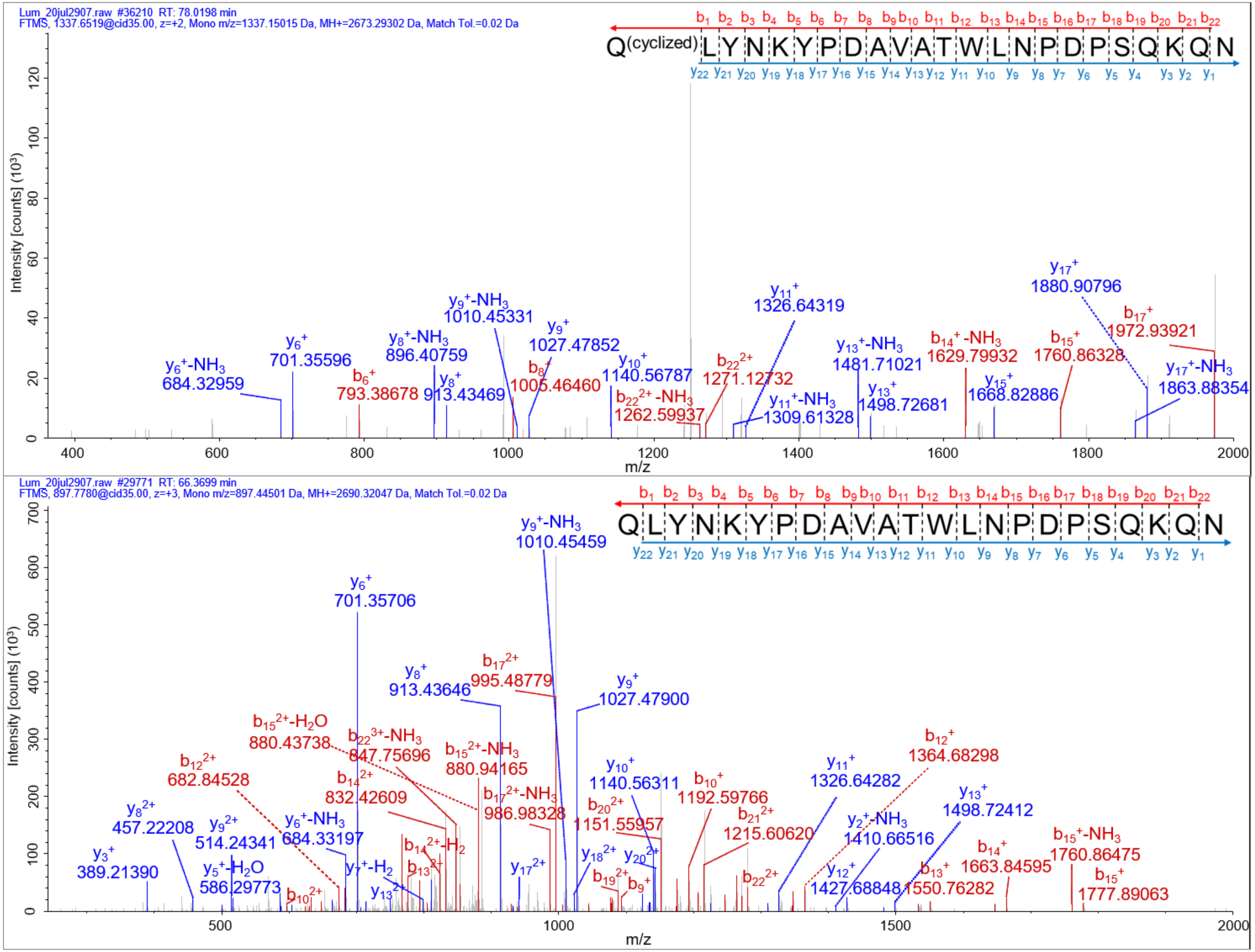

Lum_20jul2907.raw #36210 RT: 78.0198 min
FTMS, 1337.6519@cid35.00, z=+2, Mono m/z=1337.15015 Da, MH+=2673.29302 Da, Match Tol.=0.02 Da
Q(cyclized) L Y N K Y P D A V A T W L N P D P S Q K Q N
Intensity [counts] (10³)
m/z
y17+ 1880.90796
b17+ 1972.93921
y11+ 1326.64319
y9+-NH3 1010.45331
y9+ 1027.47852
y8+-NH3 896.40759
y6+ 701.35596
y6+-NH3 684.32959
b6+ 793.38678
y8+ 913.43469
b8+ 1005.46460
y10+ 1140.56787
b22 2+ 1271.12732
b22 2+ -NH3 1262.59937
y11+-NH3 1309.61328
y13+-NH3 1481.71021
y13+ 1498.72681
b14+ -NH3 1629.79932
y15+ 1668.82886
b15+ 1760.86328
y17+-NH3 1863.88354
Lum_20jul2907.raw #29771 RT: 66.3699 min
FTMS, 897.7780@cid35.00, z=+3, Mono m/z=897.44501 Da, MH+=2690.32047 Da, Match Tol.=0.02 Da
Q L Y N K Y P D A V A T W L N P D P S Q K Q N
y9+-NH3 1010.45459
y6+ 701.35706
y8+ 913.43646
b17 2+ 995.48779
y9+ 1027.47900
b15 2+-H2O 880.43738
b22 3+-NH3 847.75696
b15 2+-NH3 880.94165
b12 2+ 682.84528
b14 2+ 832.42609
b17 2+-NH3 986.98328
y10+ 1140.56311
b10+ 1192.59766
y11+ 1326.64282
b12+ 1364.68298
y13+ 1498.72412
y8 2+ 457.22208
y9 2+ 514.24341
y6+-NH3 684.33197
b20 2+ 1151.55957
b21 2+ 1215.60620
y2+-NH3 1410.66516
b15+-NH3 1760.86475
y3+ 389.21390
y5+-H2O 586.29773
y7+-H2
b14 2+-H2
b13 2+
y17 2+
y18 2+
y20 2+
b14+ 1663.84595
b10 2+
y13 2+
b19 2+
b9+
b22 2+
y12+ 1427.68848
b13+ 1550.76282
b15+ 1777.89063

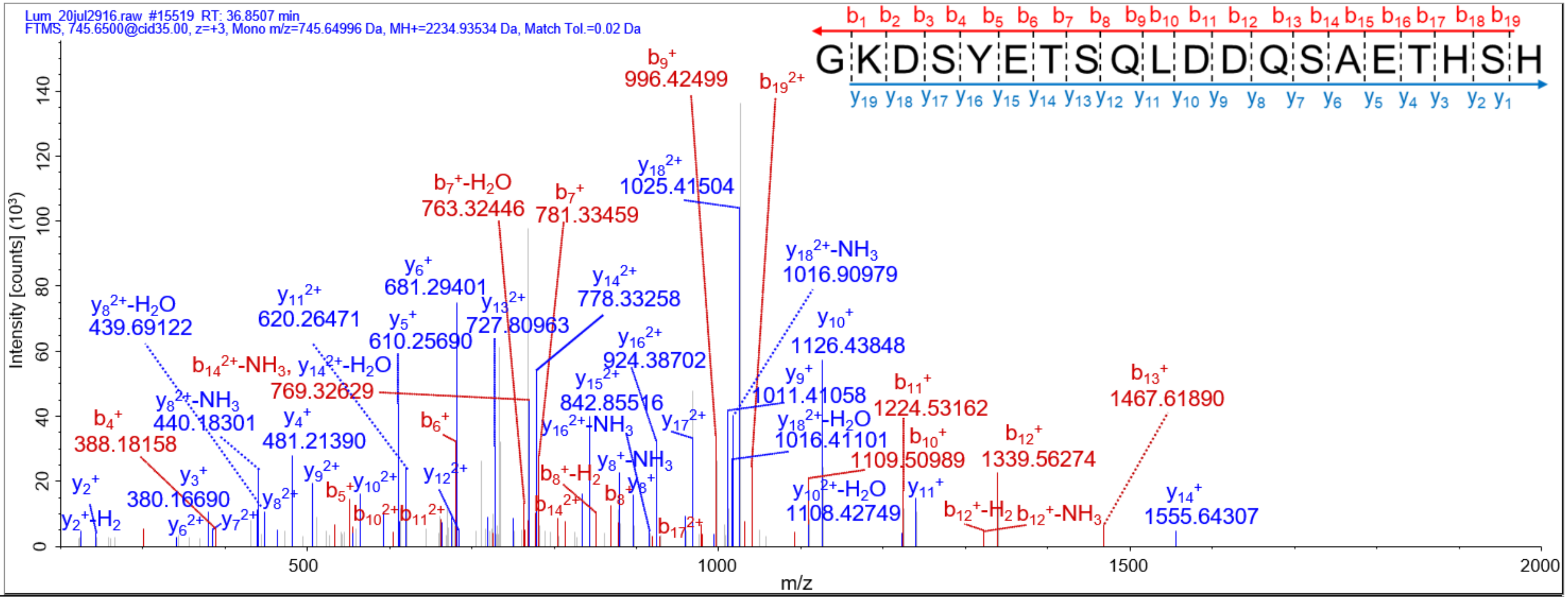

Lum_20jul2916.raw #15519 RT: 36.8507 min
FTMS, 745.6500@cid35.00, z=+3, Mono m/z=745.64996 Da, MH+=2234.93534 Da, Match Tol.=0.02 Da
G K D S Y E T S Q L D D Q S A E T H S H
b1 b2 b3 b4 b5 b6 b7 b8 b9 b10 b11 b12 b13 b14 b15 b16 b17 b18 b19
y19 y18 y17 y16 y15 y14 y13 y12 y11 y10 y9 y8 y7 y6 y5 y4 y3 y2 y1
Intensity [counts] (10³)
m/z
b9+ 996.42499
b19 2+
y18 2+ 1025.41504
b7+-H2O 763.32446
b7+ 781.33459
y18 2+-NH3 1016.90979
y6+ 681.29401
y14 2+ 778.33258
y8 2+-H2O 439.69122
y11 2+ 620.26471
y13 2+ 727.80963
y5+ 610.25690
y10+ 1126.43848
y16 2+ 924.38702
b14 2+-NH3, y14 2+-H2O 769.32629
y15 2+ 842.85516
y9+ 1011.41058
b11+ 1224.53162
b13+ 1467.61890
y8 2+-NH3 440.18301
b4+ 388.18158
y4+ 481.21390
b6+
y16 2+-NH3
y17 2+
y18 2+-H2O 1016.41101
b10+ 1109.50989
b12+ 1339.56274
y3+ 380.16690
y9 2+
y12 2+
b8+-H2
y8+-NH3
y2+
b5+
y10 2+
y8 2+
y8+
b8+
y10 2+-H2O 1108.42749
y11+
b12+-H2
b12+-NH3
y14+ 1555.64307
y2+-H2
y6 2+
y7 2+
b10 2+
b11 2+
b14 2+
b17 2+
500
1000
1500
2000
0
20
40
60
80
100
120
140

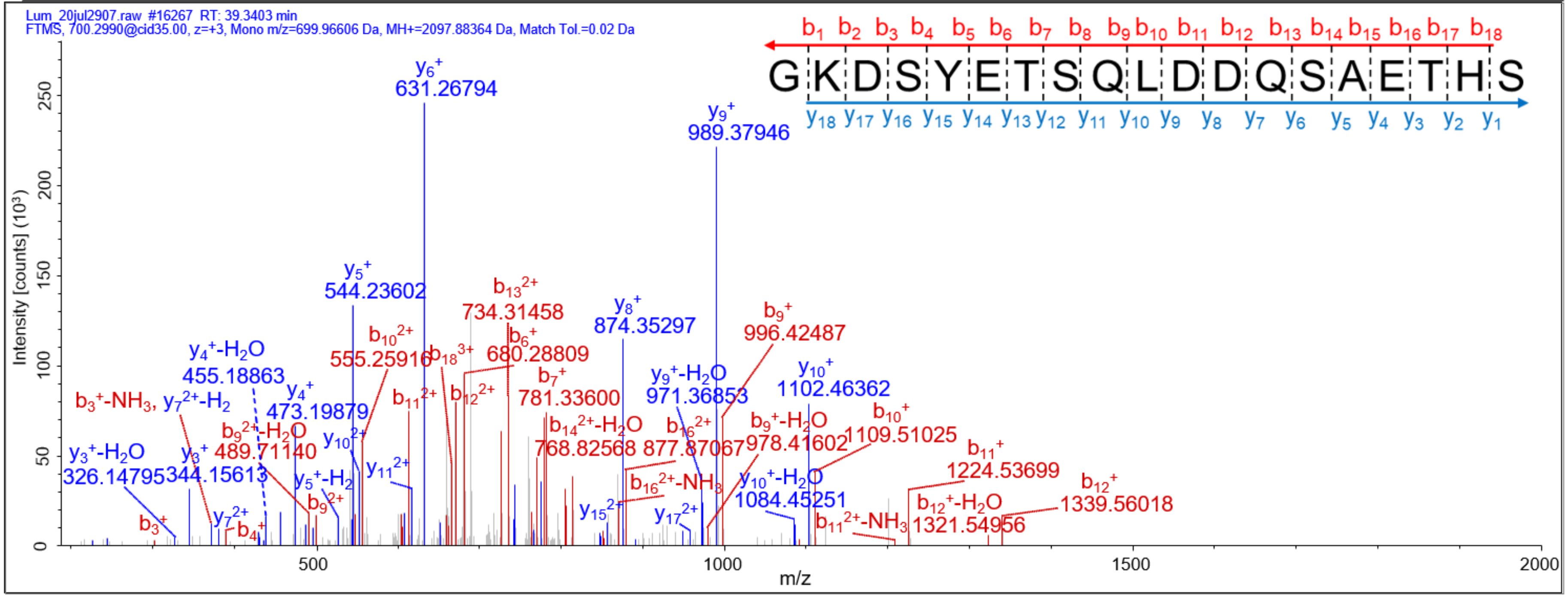

Lum_20jul2907.raw #16267 RT: 39.3403 min
FTMS, 700.2990@cid35.00, z=+3, Mono m/z=699.96606 Da, MH+=2097.88364 Da, Match Tol.=0.02 Da
G K D S Y E T S Q L D D Q S A E T H S
b1 b2 b3 b4 b5 b6 b7 b8 b9 b10 b11 b12 b13 b14 b15 b16 b17 b18
y18 y17 y16 y15 y14 y13 y12 y11 y10 y9 y8 y7 y6 y5 y4 y3 y2 y1
Intensity [counts] (10³)
m/z
y6+ 631.26794
y9+ 989.37946
y5+ 544.23602
b13 2+ 734.31458
y8+ 874.35297
b9+ 996.42487
b10 2+ 555.25916
b18 3+
b6+ 680.28809
y4+-H2O 455.18863
y9+-H2O 971.36853
y10+ 1102.46362
b7+ 781.33600
b3+-NH3, y7 2+-H2
y4+ 473.19879
b11 2+
b12 2+
b14 2+-H2O 768.82568
b16 2+ 877.87067
b9+-H2O 978.41602
b10+ 1109.51025
b9 2+-H2O 489.71140
y10 2+
y3+-H2O 326.14795
y3+ 344.15613
y11 2+
b11+ 1224.53699
b16 2+-NH3
y10+-H2O 1084.45251
b12+ 1339.56018
y5+-H2
b9 2+
b12+-H2O 1321.54956
y15 2+
y17 2+
b11 2+-NH3
b3+
y7 2+
b4+
500
1000
1500
2000
0
50
100
150
200
250

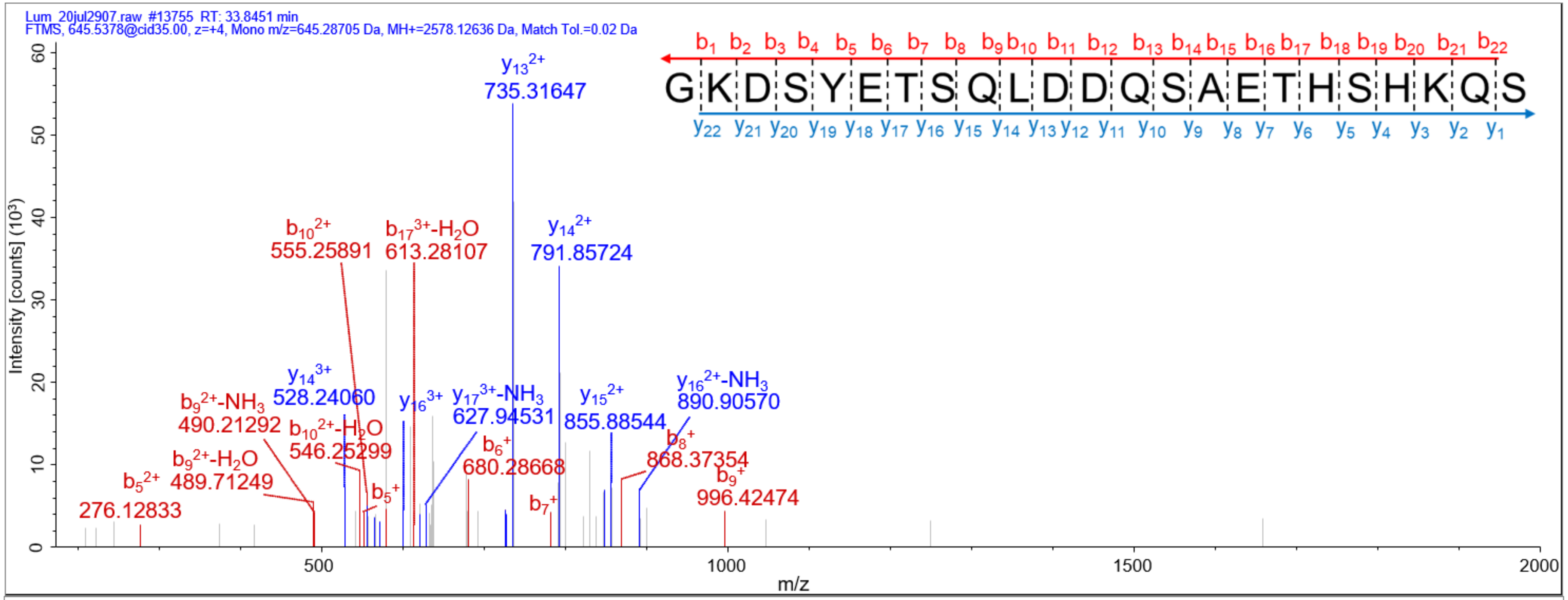
Lum_20jul2907.raw #13755 RT: 33.8451 min
FTMS, 645.5378@cid35.00, z=+4, Mono m/z=645.28705 Da, MH+=2578.12636 Da, Match Tol.=0.02 Da
b1 b2 b3 b4 b5 b6 b7 b8 b9 b10 b11 b12 b13 b14 b15 b16 b17 b18 b19 b20 b21 b22
G K D S Y E T S Q L D D Q S A E T H S H K Q S
y22 y21 y20 y19 y18 y17 y16 y15 y14 y13 y12 y11 y10 y9 y8 y7 y6 y5 y4 y3 y2 y1
Intensity [counts] (10³)
m/z
y13 2+ 735.31647
b10 2+ 555.25891
b17 3+-H2O 613.28107
y14 2+ 791.85724
y14 3+ 528.24060
y16 3+
y17 3+-NH3 627.94531
y15 2+ 855.88544
y16 2+-NH3 890.90570
b9 2+-NH3 490.21292
b10 2+-H2O 546.25299
b6+ 680.28668
b8+ 868.37354
b9 2+-H2O 489.71249
b5 2+ 276.12833
b5+
b7+
b9+ 996.42474
0 10 20 30 40 50 60
500 1000 1500 2000

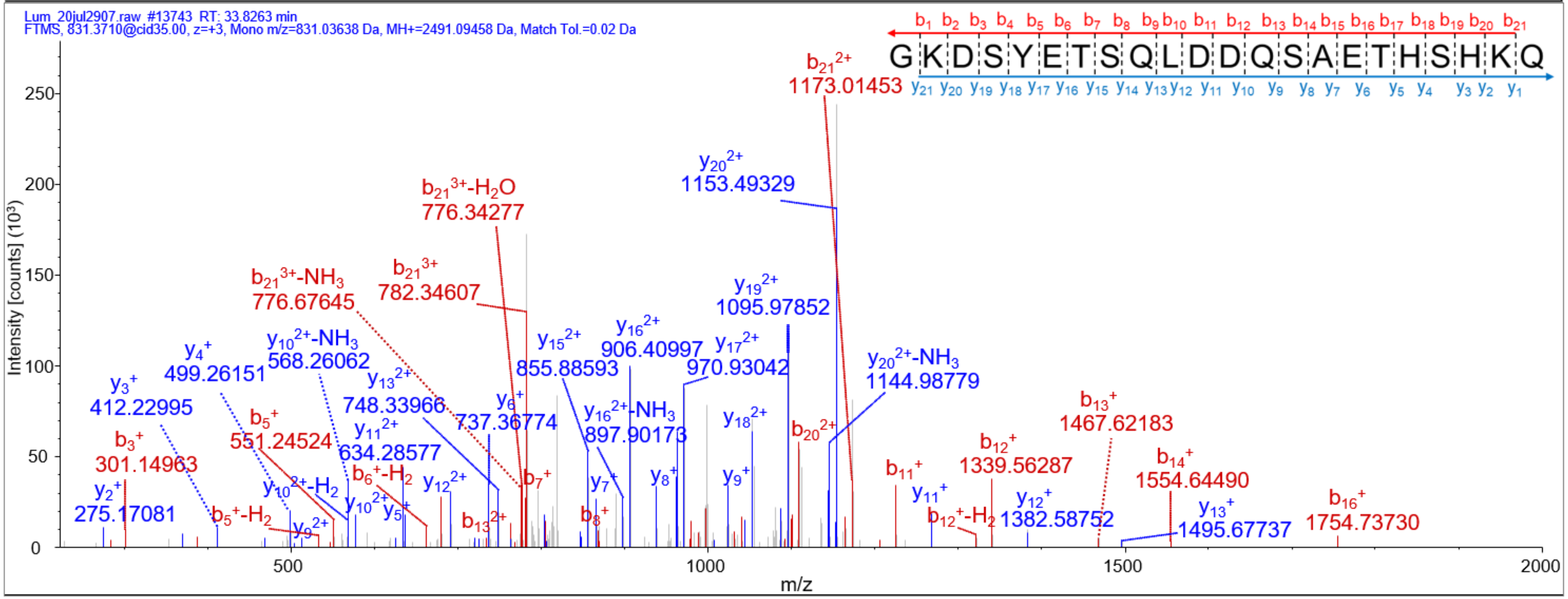
Lum_20jul2907.raw #13743 RT: 33.8263 min
FTMS, 831.3710@cid35.00, z=+3, Mono m/z=831.03638 Da, MH+=2491.09458 Da, Match Tol.=0.02 Da
b1 b2 b3 b4 b5 b6 b7 b8 b9 b10 b11 b12 b13 b14 b15 b16 b17 b18 b19 b20 b21
G K D S Y E T S Q L D D Q S A E T H S H K Q
y21 y20 y19 y18 y17 y16 y15 y14 y13 y12 y11 y10 y9 y8 y7 y6 y5 y4 y3 y2 y1
Intensity [counts] (10³)
m/z
b21 2+ 1173.01453
y20 2+ 1153.49329
b21 3+-H2O 776.34277
b21 3+-NH3 776.67645
b21 3+ 782.34607
y19 2+ 1095.97852
y16 2+ 906.40997
y17 2+ 970.93042
y15 2+ 855.88593
y20 2+-NH3 1144.98779
y4+ 499.26151
y10 2+-NH3 568.26062
y3+ 412.22995
y13 2+ 748.33966
y6+ 737.36774
y16 2+-NH3 897.90173
y18 2+
b5+ 551.24524
y11 2+ 634.28577
b3+ 301.14963
b20 2+
b13+ 1467.62183
b12+ 1339.56287
b14+ 1554.64490
b11+
y11+
y12+ 1382.58752
y13+ 1495.67737
b16+ 1754.73730
b12+-H2
y2+ 275.17081
y10 2+-H2
b6+-H2
y12 2+
b7+
y7+
y8+
y9+
y10 2+
y5+
b5+-H2
y9 2+
b13 2+
b8+
0 50 100 150 200 250
500 1000 1500 2000

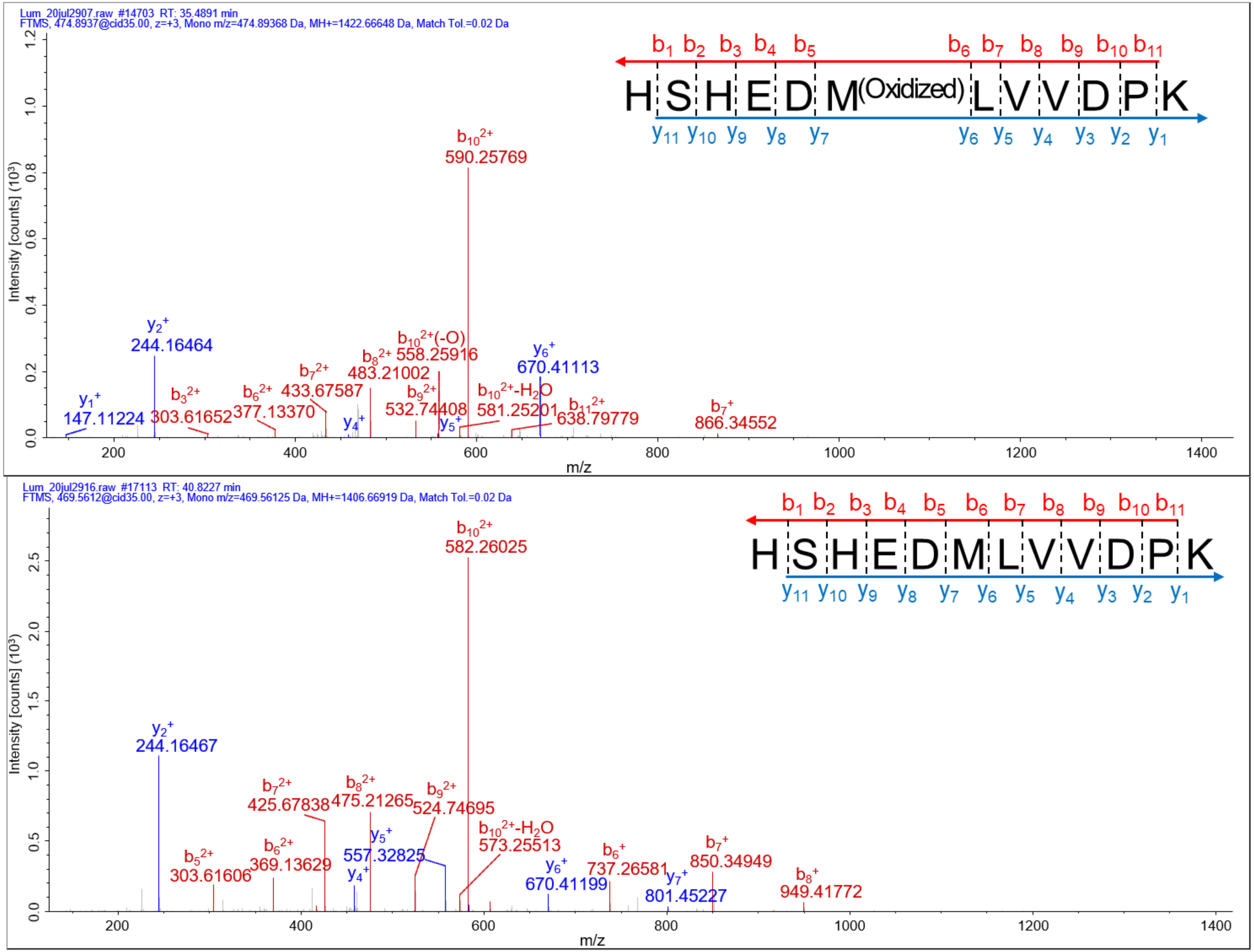
Lum_20jul2907.raw #14703 RT: 35.4891 min
FTMS, 474.8937@cid35.00, z=+3, Mono m/z=474.89368 Da, MH+=1422.66648 Da, Match Tol.=0.02 Da
Intensity [counts] (10³)
m/z
HSHEDM(Oxidized)LVVDPK
b1 b2 b3 b4 b5 b6 b7 b8 b9 b10 b11
y11 y10 y9 y8 y7 y6 y5 y4 y3 y2 y1
b10 2+ 590.25769
y2 + 244.16464
y1 + 147.11224
b3 2+ 303.61652
b6 2+ 377.13370
b7 2+ 433.67587
b8 2+ 483.21002
b10 2+(-O) 558.25916
b9 2+ 532.74408
y4 +
y5 +
b10 2+-H2O 581.25201
y6 + 670.41113
b11 2+ 638.79779
b7 + 866.34552
Lum_20jul2916.raw #17113 RT: 40.8227 min
FTMS, 469.5612@cid35.00, z=+3, Mono m/z=469.56125 Da, MH+=1406.66919 Da, Match Tol.=0.02 Da
Intensity [counts] (10³)
m/z
HSHEDMLVVDPK
b1 b2 b3 b4 b5 b6 b7 b8 b9 b10 b11
y11 y10 y9 y8 y7 y6 y5 y4 y3 y2 y1
b10 2+ 582.26025
y2 + 244.16467
b7 2+ 425.67838
b8 2+ 475.21265
b9 2+ 524.74695
b5 2+ 303.61606
b6 2+ 369.13629
y5 + 557.32825
y4 +
b10 2+-H2O 573.25513
y6 + 670.41199
b6 + 737.26581
y7 + 801.45227
b7 + 850.34949
b8 + 949.41772

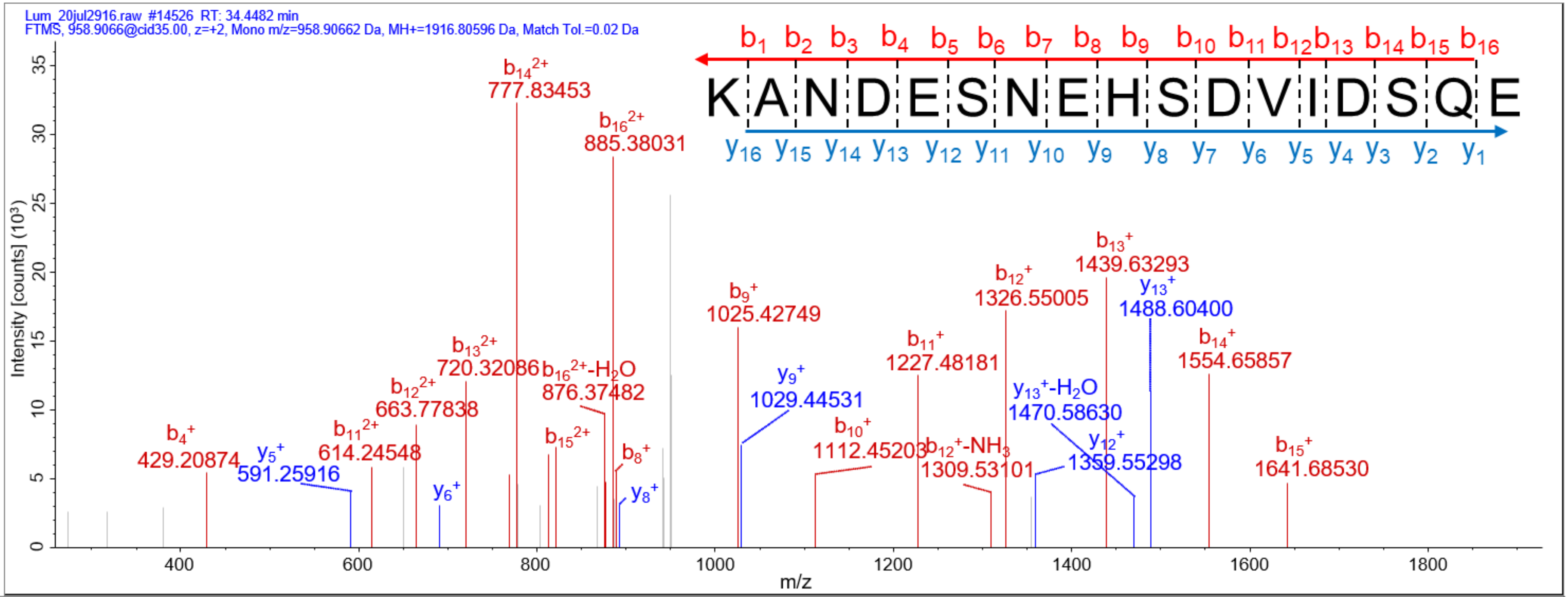
Lum_20jul2916.raw #14526 RT: 34.4482 min
FTMS, 958.9066@cid35.00, z=+2, Mono m/z=958.90662 Da, MH+=1916.80596 Da, Match Tol.=0.02 Da
K A N D E S N E H S D V I D S Q E
b_1 b_2 b_3 b_4 b_5 b_6 b_7 b_8 b_9 b_{10} b_{11} b_{12} b_{13} b_{14} b_{15} b_{16}
y_{16} y_{15} y_{14} y_{13} y_{12} y_{11} y_{10} y_9 y_8 y_7 y_6 y_5 y_4 y_3 y_2 y_1
b_{14}^{2+} 777.83453
b_{16}^{2+} 885.38031
b_{13}^{2+} 720.32086
b_{16}^{2+}-H_2O 876.37482
b_{12}^{2+} 663.77838
b_{11}^{2+} 614.24548
b_4^+ 429.20874
y_5^+ 591.25916
y_6^+
b_{15}^{2+}
b_8^+
y_8^+
b_9^+ 1025.42749
y_9^+ 1029.44531
b_{10}^+ 1112.45203
b_{11}^+ 1227.48181
b_{12}^+ 1326.55005
b_{12}^+-NH_3 1309.53101
b_{13}^+ 1439.63293
y_{13}^+ 1488.60400
y_{13}^+-H_2O 1470.58630
y_{12}^+ 1359.55298
b_{14}^+ 1554.65857
b_{15}^+ 1641.68530
Intensity [counts] (10^3)
m/z

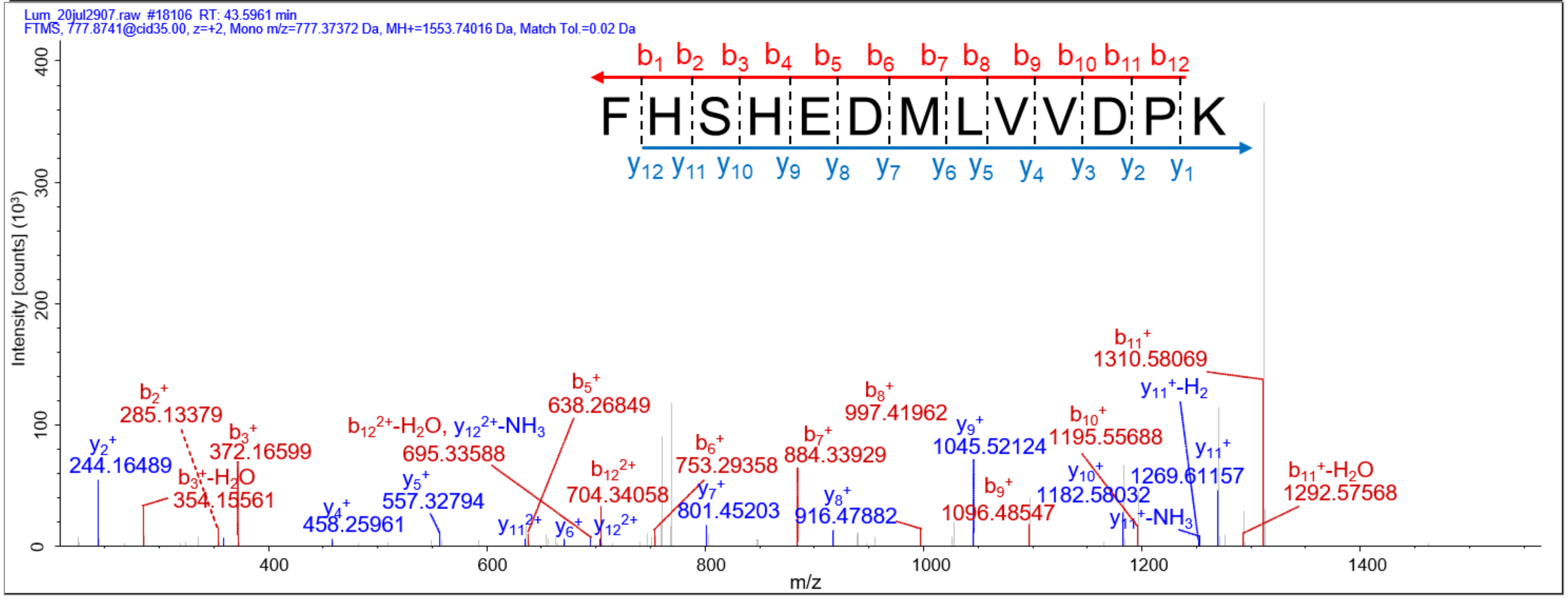
Lum_20jul2907.raw #18106 RT: 43.5961 min
FTMS, 777.8741@cid35.00, z=+2, Mono m/z=777.37372 Da, MH+=1553.74016 Da, Match Tol.=0.02 Da
F H S H E D M L V V D P K
b_1 b_2 b_3 b_4 b_5 b_6 b_7 b_8 b_9 b_{10} b_{11} b_{12}
y_{12} y_{11} y_{10} y_9 y_8 y_7 y_6 y_5 y_4 y_3 y_2 y_1
y_2^+ 244.16489
b_2^+ 285.13379
b_3^+ 372.16599
b_3^+-H_2O 354.15561
y_4^+ 458.25961
y_5^+ 557.32794
b_{12}^{2+}-H_2O, y_{12}^{2+}-NH_3 695.33588
b_5^+ 638.26849
y_{11}^{2+}
y_6^+
y_{12}^{2+}
b_{12}^{2+} 704.34058
b_6^+ 753.29358
y_7^+ 801.45203
b_7^+ 884.33929
y_8^+ 916.47882
b_8^+ 997.41962
y_9^+ 1045.52124
b_9^+ 1096.48547
b_{10}^+ 1195.55688
y_{10}^+ 1182.58032
y_{11}^+-NH_3
y_{11}^+-H_2
y_{11}^+ 1269.61157
b_{11}^+ 1310.58069
b_{11}^+-H_2O 1292.57568
Intensity [counts] (10^3)
m/z

List of Supporting Tables

Table S1: Autolytic cleavage sites in ADAMTS8 identified by LC-MS/MS. Reactions were performed for 24 h, at 37°C. Putative cleavage sites are between the residues in bold. Ratios in bold are scaled due to being only measurable in one digest.

Annotated Sequence	xCorr score	Peptide position	P1	P1'	WT/EQ ratio	z-score
[R].LQSFRPLPEPLTV**Q**.[**L**]	3.14	776-789	Q789	L790	**100**	6.7
[R].LQSFRPLPEPLTVQ**L**.[**L**]	3.15	776-790	L790	L791	**100**	6.7
[R].SHPGVQNDGNYL**A**.[**L**]	3.00	722-734	A734	L735	**100**	6.7
[K].YTFFVPNDVDFS**M**.[**Q**]	2.17	804-816	M816	Q817	**100**	6.7
[K].YTFFVPNDVDFSMQ**S**.[**S**]	2.54	804-818	S818	S819	**100**	6.7
[**A**].**L**KTADGQYLLNGNL AISAIEQDILVK.[G.]	4.87	735-760	A734	L735	**100**	6.7
[**Y**].**L**LNGNLAISAIEQDILVK.[G]	3.94	743-760	Y742	L743	**100**	6.7

Table S2: List of differentially regulated genes encoding secreted/ECM proteins in pulmonary artery hypertension (PAH). Only ECM/secreted protein-encoding genes with changes in expression compared with non-PAH controls≥1 are reported. GEO, Gene Expression Omnibus; PAECs, Pulmonary artery endothelial cells; SMCs, smooth muscle cells.

**Reference (GEO ID)**	**RNA source**	**Platform**	**NCBI gene name**	**Up/down-Regulated (Fold)**
65	Lungs from PAH patients	Affymetrix	*ANGP1*	Down
			GAS6	Down
			THBD	Down
66	Lungs from PAH patients	Agilent	*AGRP*	Up (1.1)
			C1QA	Up (1.0)
			COLQ	Down (1.4)
			FRZB	Down (1.6)
			GREM1	Down (1.6)
			IL16	Down (1.9)
			LCAT	Down (1.2)
			MATN2	Down (1.6)
			MMP15	Up (1.3)
			NRTN	Up (2.3)
			NTN3	Up (1.2)
			PLA2G12B	Up (1.5)
			PRSS8	Up (1.2)
			SERPINA5	Down (1.4)
			TGFB3	Down (1.2)
			TNC	Down (1)
			VEGFA	Up (1.4)
67	Lungs from PAH patients	Agilent	*COL1A2*	Up (1.0)
			COL3A1	Up (1.1)
			COL4A1	Up (1.0)
			COL6A3	Up (1.1)
			IGFBP4	Up (1.0)
			MGP	Up (1.1)
			SPARC	Up (1.1)
			SPOCK2	Up (1.0)
			TGFB1	Up (1.0)
			THBS1	Up (1.0)
			TNFAIP3	Up (2.1)
			VIM	Up (1.2)
			VWF	Up (1.1)
68 (GSE24988)	Lungs from PAH patients	Affymetrix	*FGF4*	Up
			MMP1	Up
			MMP7	Up
			MMP13	Up
			SPP1	**Up**
69 (GSE53408)	Lungs from PAH patients	Affymetrix	*POSTN*	Up (1.5)
70	PAH PAECs	Illumina	*COL4A1*	Down (1.7)
			COL4A2	Down (1.4)
			LOX	Down (1.1)
			TIMP1	Down (1.1)

Table S2 (continued)

Reference (GEO ID)	RNA source & comparison	Platform	NCBI gene name	Up/down-Regulated (Fold)
71	SMCs from PAH patients	Affymetrix	*ADAMTS5*	Down (2.6)
			BMP4	Up (3.3)
			CST1	Down (7.0)
			CTSH	Up (2.3)
			EREG	Down (6.3)
			GDF15	Down (1.9)
			MXRA5	Down (7.2)
			NID2	Down (2.5)
			SMOC1	Up (3.3)
			SOD3	Down (1.6)
			VEGFA	Down (1.7)
33	Senescent PAH-SMC	Affymetrix	*ADAMTS8*	Down
			CLEC3B	Down
			COL14A1	Down
			COL15A1	Down
			KAL1	Down
			MMP11	Down
			MMP16	Down
			NCAM1	Up (4)*
			SPP1	**Up (14)***
			TNC	Up (9)*
			TGFB1	Up (10)*
			VCAM1	Down
			VTN	Down
72	PAH fibroblasts	Affymetrix	*Adam23*	Down (2.5)
			ADAMTS19	Down (2.3)
			BGN	Up (2.4)
			BMP6	Down (2.2)
			EPGN	Down (2.7)
			RELN	Down (4.8)
			SERPINE2	Down (3.6)
			SERPINI1	Down (2.5)
			SPON2	Up (2.2)
			TNC	Up (3.3)
			VCAN	Down (2.8)
73 (GSE85618)	Lungs from rats (hypoxia model)	Affymetrix	*CXCL5*	Down (1.9)
			CLEC4D	Up (2.7)
			CSF3R	Up (2.4)
			GREM1	Down (2.2)
			MMP8	Up (3.9)
			RETNLG	Up (3.3)
			S100A8	Up (3.4)
			S100a9	Up (3.1)
			THBS2	Down (2.0)

*estimated from Figure 1

Table S2 (continued)

Reference (GEO ID)	RNA source & comparison	Platform	NCBI gene name	Up/down-Regulated (Fold)
74 (GSE117261)	Lungs from PAH patients	Affymetrix	*ADAMTS17*	Up (1.1)
			ADAMTS1L	Up (1.4)
			ASPN	Up (3.3)
			BMP6	Up (2.1)
			CFD	Up (1.7)
			COL14A1	Up (2.4)
			COL18A1	Up (1.2)
			COL4A3	Up (1.4)
			COL6A6	Up (2.1)
			CXCL12	Up (2.0)
			ECM2	Up (1.9)
			EFEMP2	Up (1.4)
			IGFBP7	Up (1.4)
			LTBP1	Up (2.0)
			LTBP2	Up (1.9)
			LTBP3	Up (1.4)
			LTBP4	Up (1.5)
			LUM	Up (1.6)
			MASP1	Up (1.1)
			MATN2	Up (1.5)
			METTL24	Up (1.2)
			MFAP2	Up (1.5)
			NAALADL2	Up (1.3)
			OGN	Up (2.2)
			PAMR1	Up (1.6)
			POSTN	Up (2.9)
			TNXB	Up (1.5)
			COL4A1	Up (1.2)
			COL4A2	Up (1.2)
			LOX	Up (1.2)
			TGFB2	Up (1.3)
37	Lungs from PAH patients	Affymetrix	*PI15*	Up (4.3)
			POSTN	Up (5.3)
			SPP1	**Up (3.6)**
75	Lungs from rats (hypoxia model)	Illumina	*COL4A1*	Up (1.9)
			FN1	Up (1.3)
			SPP1	**Up (1.3)**
			THBS4	Up (1.3)
			TNC	Up (1.5)
			VTN	Up (1.3)

Table S3: List of semi-tryptic peptides informing assignment of putative ADAMTS8 cleavage sites in OPN. Putative cleavage sites are between the residues in bold, note that the other end of each peptide is tryptic. A ratio of "100" indicates a peptide found only in the WT digests, and a ratio of "0.01" indicates a peptide found only in the EQ digest. # indicates amino acid cyclization (Q), † indicates amino acid oxidation (M).

Digest Time	Annotated sequence	XCorr score	Peptide position	P1	P1'	WT/EQ ratio	z - score
2 h	[**D**].**D**QSAETHSHK	2.06	218-227	D217	D218	100	2.87
2 h	GKDSYETSQLDDQSAETHSHK**Q**.[**S**]	4.87	207-228	Q228	S229	100	2.69
2 h	#QLYNKYPDAVATWLNPDPSQKQ**N**.[**L**]	7.65	31-53	N53	L54	100	2.87
2 h	QLYNKYPDAVATWLNPDPSQKQ**N**.[**L**]	2.37	31-53	N53	L54	100	2.87
2 h	[**Q**].**S**AETHSHK	2.86	220-227	Q219	S220	0.01	3.73
24 h	[**D**].**D**QSAETHSHK	2.1	218-227	D217	D218	100	2.69
24 h	[**E**].**F**HSHEDMLVVDPK	4.09	264-276	E263	F264	100	2.69
24 h	GKDSYETSQLDDQSAETH**S**.[**H**]	7.84	207-225	S225	H226	100	2.69
24 h	GKDSYETSQLDDQSAETHS**H**.[**S**]	7.25	207-226	H226	S227	100	2.69
24 h	GKDSYETSQLDDQSAETHSHKQ**S**.[**R**]	2.61	207-229	S229	R230	100	2.69
24 h	[**F**].**H**SHED†MLVVDPK	2.61	265-276	F264	H265	100	2.69
24 h	[**F**].**H**SHEDMLVVDPK	3.97	265-276	F264	H265	100	2.69
24 h	KANDESNEHSDVIDSQ**E**.[**L**]	3.08	235-251	E251	L252	100	2.69
24 h	[**Y**].**N**KYPDAVATWLNPDPSQK	5.91	34-51	Y33	N34	100	2.69
24 h	#QLYNKYPDAVATWLNPDPSQKQ**N**.[**L**]	5.37	31-53	N53	L54	100	2.69
24 h	QLYNKYPDAVATWLNPDPSQKQ**N**.[**L**]	7.51	31-53	N53	L54	100	2.69
24 h	GKDSYETSQLDDQSAETHSHK**Q**.[**S**]	7.18	207-228	Q228	S229	45.343	2.15
24 h	[**A**].**I**PVKQADSGSSEEKQLYNK	4.08	17-35	A16	I17	0.01	3.63
24 h	QLYNKYPDAVATWLNPD.[**P**]	2.82	31-47	D47	P48	0.01	3.63
